# A non-invasive method for concurrent detection of early-stage women-specific cancers

**DOI:** 10.1038/s41598-022-06274-9

**Published:** 2022-02-10

**Authors:** Ankur Gupta, Ganga Sagar, Zaved Siddiqui, Kanury V. S. Rao, Sujata Nayak, Najmuddin Saquib, Rajat Anand

**Affiliations:** 1 PredOmix Technologies Private Limited, Tower B, SAS Tower, Medicity, Sector – 38, Gurugram, 122002 India; 2 PredOmix, Inc., 9853 Pacific Heights Blvd., San Diego, CA 92121-4721 USA

**Keywords:** Machine learning, Diagnostic markers, Metabolomics, Molecular medicine, Cancer, Cancer screening

## Abstract

We integrated untargeted serum metabolomics using high-resolution mass spectrometry with data analysis using machine learning algorithms to accurately detect early stages of the women specific cancers of breast, endometrium, cervix, and ovary across diverse age-groups and ethnicities. A two-step approach was employed wherein cancer-positive samples were first identified as a group. A second multi-class algorithm then helped to distinguish between the individual cancers of the group. The approach yielded high detection sensitivity and specificity, highlighting its utility for the development of multi-cancer detection tests especially for early-stage cancers.

## Introduction

Cancer remains one of the most pervasive causes of death worldwide, and the incidence continues to rise globally^1–3^. In females, cancer is the second most important cause of death, with about 7 million new cases, and over 3.5 million deaths, being recorded each year^4^. The leading female-specific cancers are breast cancer, cervical cancer, uterine or endometrial cancer, and ovarian cancer^4,5^. Of these, breast cancer is the most frequently diagnosed and accounts for 25% of cancer cases, along with 15% of cancer-related deaths among women across the world^6^. In comparison, cervical cancer is the fourth most frequently diagnosed cancer in women, with an estimate of over 500,000 cases worldwide^7^. Endometrial cancer accounts for about 5% and 2% of global cancer incidence and mortality among women^8^, whereas ovarian cancer accounts for about 4% of the women cancers^9^.

Poor prognosis in female-specific cancers is often a result of late-stage presentation, with additional factors such as diagnostic uncertainties and/or diagnostic errors also contributing^10–12^. Significant improvements in treatment outcome can, however, be accomplished if the cancer is accurately detected at the earliest possible stage. This ensures that cures are more achievable, and the treatment is less morbid^13^. Unfortunately, though, effective screening paradigms exist only for a restricted subset of cancers and these include colonoscopy^14^, prostate specific antigen^15^, mammography^16^, and cervical cytology^17^. However, the impact of these tests too has been limited because the efficacy of some of them remain questionable ^18^, and also the fact that many patients fail to comply with screening guidelines^19^. Similarly, biomarkers based either on DNA or proteins have also not yet yielded accurate tests for early-stage cancer detection. Low penetrance in the risk groups, and/or low concentrations of the cancer markers, have proven to be the confounding factors in this regard^20–24^. Indeed, diagnoses for most cancers are still prompted by symptoms that only become apparent at the later stages. As a result, development of effective, non-invasive, screening methods for early cancer detection remains one of the foremost challenges facing modern cancer research.

The last decade has seen considerable interest in employing ‘omics’-based approaches for early-stage cancer diagnosis. Attempts have been made to capture cancer-specific molecular alterations though interrogation of either the genome, epigenome, transcriptome, proteome, metabolome, or the lipidome. Of these approaches, metabolomics best reflects changes in phenotype and—therefore—offers the most promising possibilities for translation to clinical application^25^. Metabolites represent proximal reporters of disease, and the idea that the metabolite composition of biological fluids reflects the health of an individual is now generally accepted^26^. The application of metabolomics for cancer diagnosis is especially relevant given that cancers are known to possess unique metabolic phenotypes due to altered metabolism. Consequently, the patterns of metabolites that are produced likely encapsulate ‘signatures’ that correlate with either emergence, presence, or behavior of a cancer^25^. Using biological fluids such as urine, saliva, or serum, metabolite biomarkers have been identified for several cancers^27^. For example, mass spectrometric studies have identified metabolite changes that are characteristic of breast cancer in blood of patients^28,29^. Similarly, studies have revealed that metabolomics of saliva and urine may also be used to distinguish cancer patients from healthy individuals^30^.

Despite these recent advances in metabolomics-based cancer detection, however, reliable methodologies for accurate diagnosis of early-stage disease are still lacking. In this context, the majority of studies have focused on identifying discrete metabolites, or sets of discrete metabolites, that are either up- or down-regulated in a specific cancer. What has been less explored are approaches that treat metabolome data as analog outputs, from which patterns that characterize a given disease—and its stage—can be extracted. Untargeted metabolomics by liquid chromatography coupled with mass spectrometry (LC–MS) provides for maximal coverage of metabolite species in a sample^31,32^. While the resulting data is complex it is, nonetheless, very information rich^33^. Such data is readily amenable to analysis using pattern recognition algorithms and, therefore, has the potential for accurately diagnosing the health state of an individual.

In the present report, we describe an integrated method for the simultaneous detection of early stages of the four most prominent women-specific cancers. These cancers are breast cancer, endometrial cancer, cervical cancer, and ovarian cancer (BECO). Our method combines untargeted serum metabolomics with data analysis using a machine learning algorithm to capture the complex metabolite signatures that specifically characterize early stages of the individual cancers. The detection accuracy obtained with this method is significantly superior to that of other existing methods. Additionally, it enables simultaneous screening for all the four cancers in a single analysis.

## Results

### Characteristics of samples employed for the study

Table 1 details the sample set employed for the present study. The number of samples for each of the target cancers is shown, along with the number of normal control samples. Majority of the samples were from women between the ages of 30–80 years, although a few samples from women in the 20 to 30- and 81 to 90-year age groups were also included (Table 1). Donors were predominantly Caucasian women (87%), with a lesser number of non-White donors, which included African American, Hispanic, and Asian women (Table 1). Thus, the cumulative number of samples employed for the present study was 1369, of which 1119 were derived from the cancers of interest (i.e. breast, endometrial, cervical and ovarian cancer), while the remainder (n = 250) were the normal controls.

**Table 1:** Demographic, ethnicity, and BMI group of the sample set used in the study.

Parameter	Individual cancers and controls				
	Demographic and clinical data				
	Normal control (n = 250)	Endometrial cancer (n = 304)	Breast cancer (n = 303)	Cervical cancer (n = 250)	Ovarian cancer (n = 262)
**Age (years)**					
20–30	18	0	2	20	6
31–40	40	6	41	79	43
41–50	92	48	80	76	72
51–60	69	156	111	48	72
61–70	21	76	53	21	60
71–80	10	14	14	6	6
81–90	0	4	2	0	3
**BMI (kg/m**^**2**^**)**					
10 to 30	119	130	270	90	86
> 30	8	172	24	22	50
**Ethnicity**					
White	153	302	246	233	256
Non-white	97	2	57	17	6
**Cancer stage**					
0	0	0	36	70	19
I	0	304	267	180	243

### Data generation and analysis

**Figure 1:**
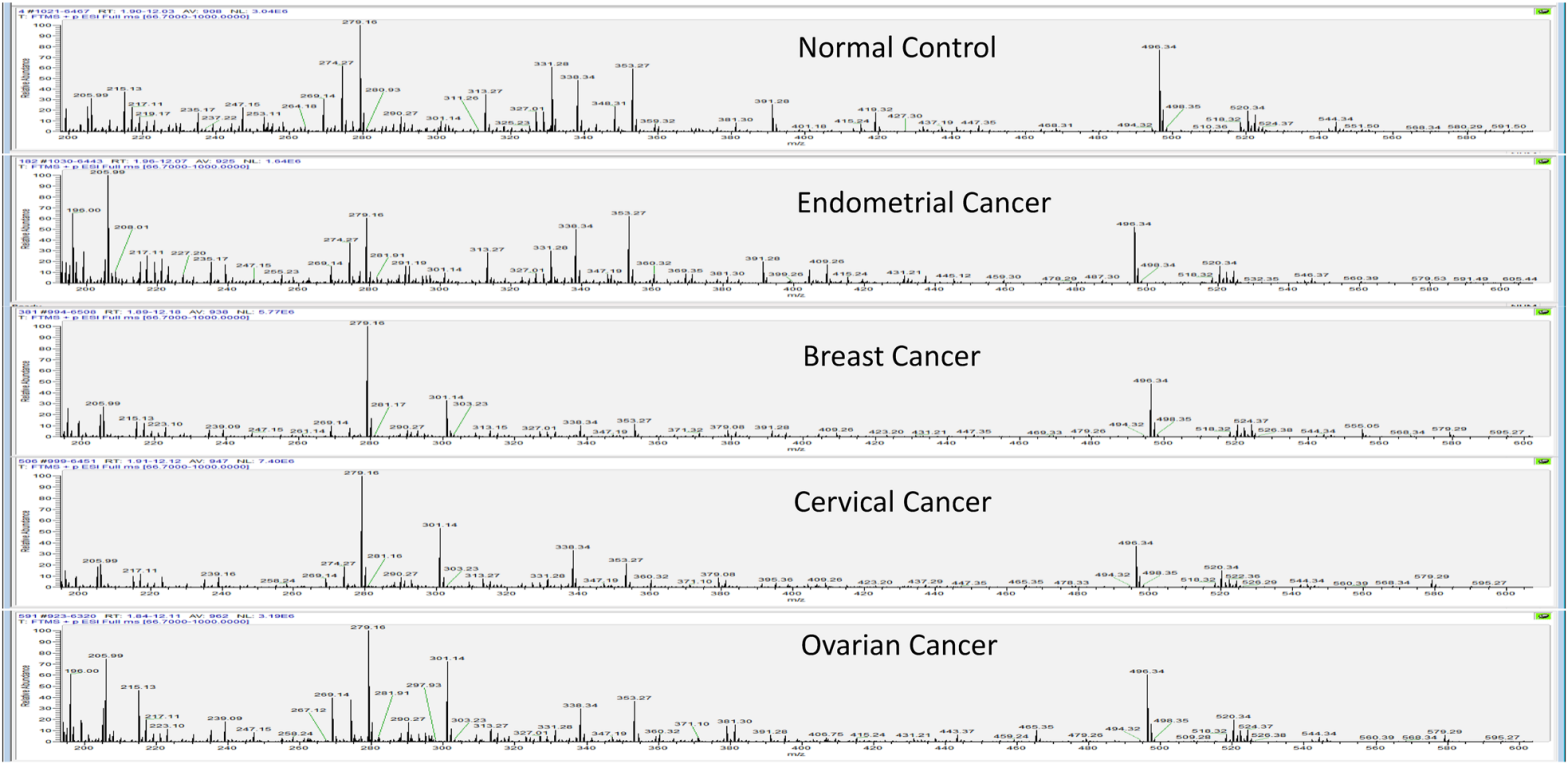
Ion chromatograms of representative samples from the normal control and the individual cancer groups. The total run time for the LC resolution was 14 min, with every sample run being alternated with a blank run. For the blank run involved injection of a 1:1 mixture of methanol and water. Comparatively, spectra in each case were following a trend with major changes seen from 200 to 600 m/z with the time ranging from 3 to 11 min.

**Figure 2:**
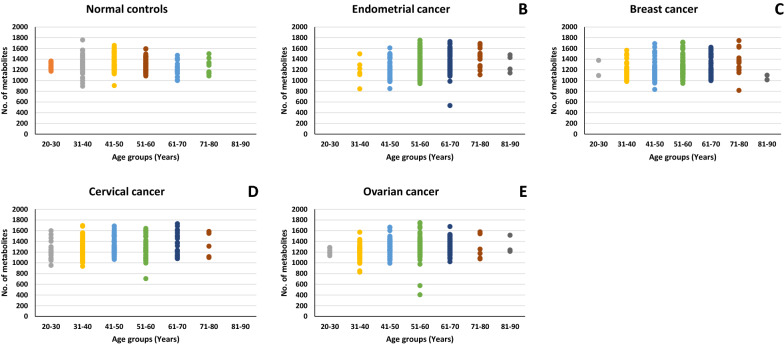
Age-wise detection of detected metabolites. Figure provides a graphical representation of the number of metabolites detected across the individual age groups, for the normal control set as well as the individual cancer groups. The cumulative unique metabolites detected in normal control samples were 5895. While, endometrial, breast, cervical and ovarian cancer samples were found to have 5971, 5982, 6300 and 6336 respectively.

**Figure 3:**
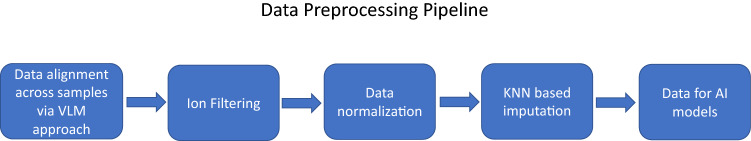
Data processing pipeline. The data preprocessing pipeline used to render the data amenable to AI modeling is depicted here (details are given in the text).

Positive ion mode UPLC-MS/MS interrogation of the serum metabolome of samples described in Table 1 resulted in the detection of > 20,000 spectral features (R_t_, m/z pairs). The untargeted metabolomics approach (Fig. 1) generated a large metabolites list, which was further divided into subset of normal control, endometrial cancer, breast cancer, cervical cancer, and ovarian cancer with 5895, 5971, 5982, 6300 and 6336 metabolites respectively. The total number of unique metabolites identified in our study was 7596 in number. Distribution of the number of unique metabolites identified in samples from the normal control, and individual cancer types, as a function of the age groups, is shown in Fig. 2. Subsequent processing of this data through our in-house pipeline, which sequentially involved normalization, gap filling, data transformation, and feature filtering and selection (“Methods”, Fig. 3), resulted in a matrix consisting of 2764 features across 1369 samples.

**Figure 4:**
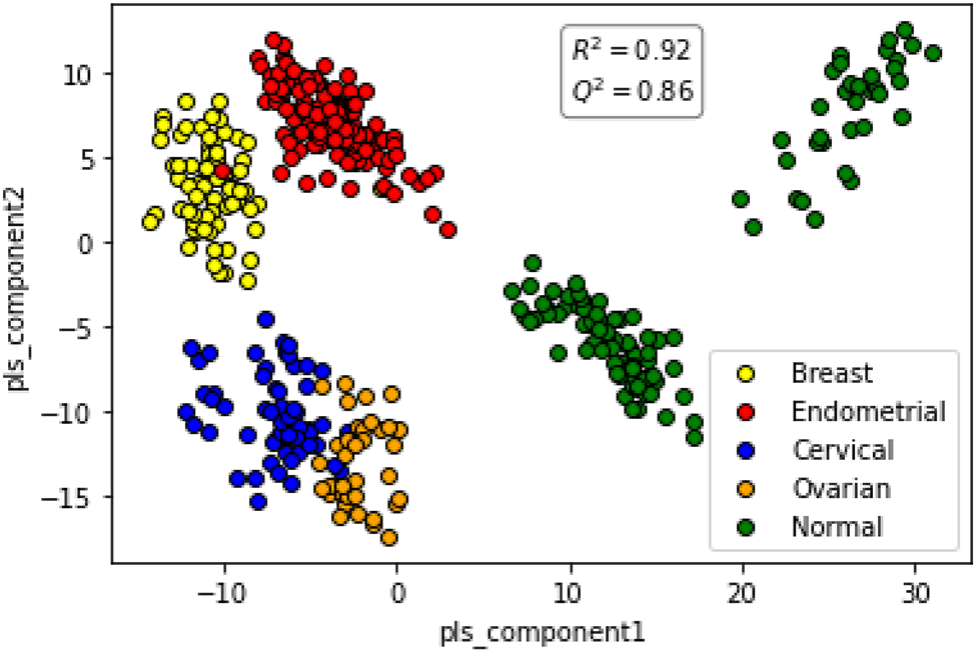
PLSDA plot distinguishes between the individual cancers and also the normal controls. Figure presents a PLSDA plot of the matrix of sample-specific metabolites versus metabolite intensity for normal controls and the individual women-specific cancer sets. The separation obtained between the individual groups is shown. The R^2^ and Q^2^ values obtained are given.

Out of total 1369 samples, 304 samples were of Endometrial Cancer, 303 Breast Cancers, 250 Cervical Cancer, 262 Ovarian Cancer and 250 Normal Control samples. To determine whether there is any difference in these samples based on metabolite data, the matrix generated above was used. A PLSDA plot was made using the matrix as shown in Fig. 4. The figure shows that cancer samples can be clearly distinguished from the normal control samples. Additionally, encouraging separation was also obtained between the individual cancer subsets (Fig. 4). To quantify how well these can be distinguished, an AI analysis was done on the data as described below to find common patterns in metabolite variations within cancer samples which is different from normal control samples. Briefly, keeping in mind clinical applications of the AI model, a layered approach was used here in which first, an AI model was developed to differentiate the breast, endometrial, cervical, and ovarian (BECO) cancers from normal controls, and then between the individual cancers.

### Distinguishing women-specific cancer samples from controls

**Figure 5:**
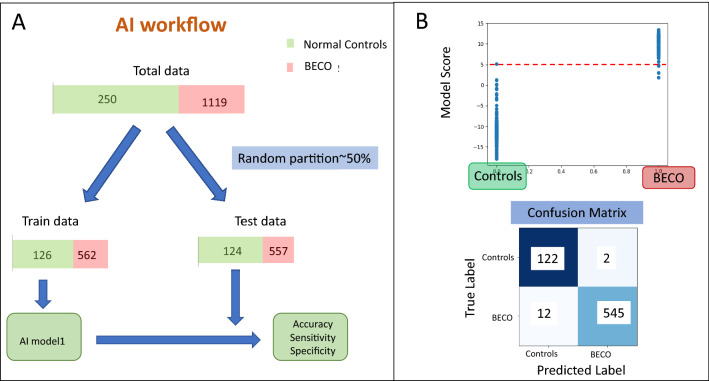
AI workflow for distinguishing BECO cancers from normal controls and its application. Panel A depicts the AI workflow employed to test the AI model for distinguishing between the women-specific cancer group (BECO) from the Normal controls. Panel B depicts the results from testing of the trained model for distinguishing women-specific cancers (BECO) from normal controls showing clear separation of disease. The separation achieved between the cancer and the control group is shown in the form of a confusion matrix, with the resulting sensitivity and specificity values also given.

To distinguish breast, endometrial, cervical, and ovarian cancers as a group (BECO cancers) from normal controls, the data was randomly partitioned into training and test datasets in comparable proportion between the individual BECO cancers and the normal controls. This resulted in 562 BECO Cancer samples and 126 Controls in training set. And 557 BECO Cancer samples and 124 Controls in test set. The AI model was applied on the training set (Supplementary Table S1, Fig. 5A) and tested in the test set to obtain Accuracy, Sensitivity and Specificity values. The logistic regression function was applied on the training data to find a function separating BECO Cancer samples versus Normal Control samples. Class balancing parameters were configured in the model to deal with the imbalance of classes in the training dataset. The trained algorithm finds a score for each of the sample according to the formulae below:

$${\text{y}}\_{\text{score}} = {\text{x}}0 + {\text{x1}}*{\text{I}}_{{1}} + {\text{x2}}*{\text{I}}_{{2}} + {\text{ x3}}*{\text{I}}_{{3}} + \cdots + {\text{x2823}}*{\text{I}}_{{{2823}}}$$

Here, × 0 is a constant number, I_i_ (1 ≤ i ≤ 2823) is the intensity of metabolite i present in the respective sample. Supplementary Figure S1 gives the value of coefficient xi (1 ≤ i ≤ 2823) for each metabolite.

The evaluation of the trained model as applied on test set for a single partition of data is shown in Fig. 5B. The scatter plot shows the Model Score for Normal Controls and BECO Cancer cases. The model scores are clearly seen to be different between Normal Controls and BECO Cancer samples where on applying a threshold of 5 to differentiate between two types results in a confusion matrix as shown in Fig. 5B. Sensitivity, Specificity and Accuracy were calculated from formulae given in “Methods”, and this resulted in Sensitivity of 98%, Specificity of 98.3%, and an Accuracy of 98%.

### Differentiating endometrial, breast, cervical, ovarian from each other

In the second step, another multiclass AI model was layered on top of the first model, which acted on the predicted cancers samples from the first model (breast, endometrial, cervical or ovary) and gave a multiclass score to each sample: one score for each disease class denoting the probability of the sample belonging to the respective disease class.

**Figure 6:**
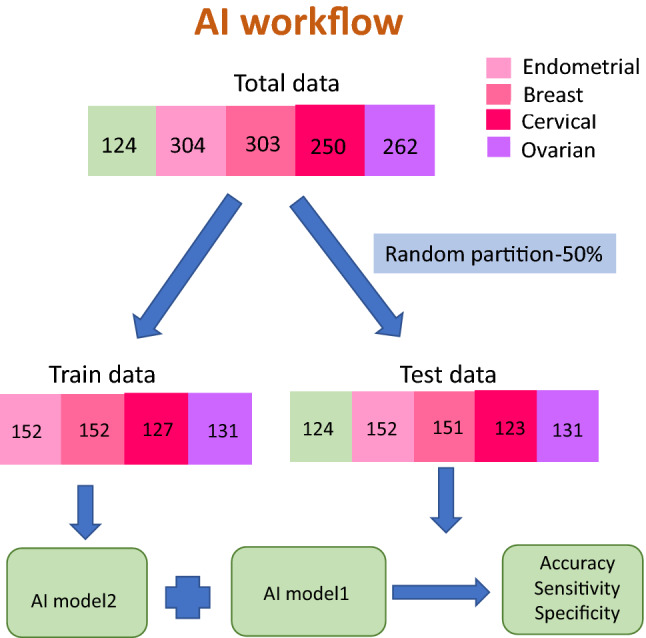
Partitioning of training and test data sets for the multiclass AI model. (**A**) shows the segregation of the individual cancer sets for training and testing of the multiclass AI model-2 (see text) for distinguishing between the individual cancers of the BECO group.

Here, out of a total of 1119 BECO samples, 304 samples were Endometrial Cancer, 303 were Breast Cancer samples, 250 were Cervical Cancer samples, and 262 were Ovarian Cancer samples. The data was randomly partitioned into training and test datasets in equal proportion as shown in Fig. 6 and Supplementary Table S2. This resulted in 152 Endometrial Cancer samples, 152 Breast Cancer samples, 127 Cervical Cancer samples and 131 Ovarian Cancer samples in the training set, and in 152 Endometrial Cancer samples, 151 Breast Cancer samples, 123 Cervical Cancer samples, and 131 Ovarian Cancer samples in the test set. In addition, a set of 124 normal control samples were added to the test set. Then, a one versus rest (OVR) classifier multiclass classification model was made using the training samples to give AI model—2. Following this, a two layered modeling scheme was applied on the test set. That is, firstly, AI model—1 differentiating BECO versus normal samples was applied on the test set. Then, AI model—2 was applied on the resulting predicted BECO samples. This resulted in four scores for each sample, with each score defining probability of the respective sample belonging to one of the four classes.

For the multi class model: AI model—2, a one versus rest (OVR) classifier multiclass classification model was made using the training samples. The trained algorithm finds 4 scores for each of the sample according to the formulae below:

$$\begin{aligned} & {\text{y}}\_{\text{score1}} = {\text{y}}0 + {\text{y1}}*{\text{I}}_{{1}} + {\text{ y2}}*{\text{I}}_{{2}} + {\text{ y3}}*{\text{I}}_{{3}} + \cdots + {\text{y2823}}*{\text{I}}_{{{2823}}} \\ & {\text{y}}\_{\text{score2}} = {\text{z}}0 + {\text{z1}}*{\text{I}}_{{1}} + {\text{ z2}}*{\text{I}}_{{2}} + {\text{ z3}}*{\text{I}}_{{3}} + \cdots + {\text{z2823}}*{\text{I}}_{{{2823}}} \\ & {\text{y}}\_{\text{score3}} = {\text{a}}0 + {\text{a1}}*{\text{I}}_{{1}} + {\text{ a2}}*{\text{I}}_{{2}} + {\text{ a3}}*{\text{I}}_{{3}} + \cdots + {\text{a2823}}*{\text{I}}_{{{2823}}} \\ & {\text{y}}\_{\text{score4}} = {\text{b}}0 + {\text{b1}}*{\text{I}}_{{1}} + {\text{ b2}}*{\text{I}}_{{2}} + {\text{ b3}}*{\text{I}}_{{3}} + \cdots + {\text{b2823}}*{\text{I}}_{{{2823}}} \\ \end{aligned}$$

Here, y0, z0, a0, b0 are constant number, I_i_ (1 ≤ i ≤ 2823) is the intensity of metabolite i present in the respective sample. Supplementary Figures S2 gives the value of coefficient yi, zi, ai, bi (1 ≤ i ≤ 2823) for each metabolite.

**Figure 7:**
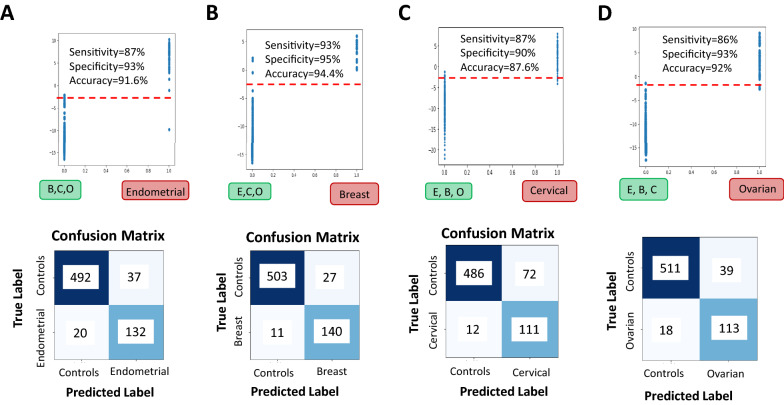
Testing the multiclass model for its ability to distinguish the individual cancer groups. Panel (**A**) shows the results of specifically testing the multiclass trained model for separation of endometrial cancer samples from the other cancers (breast, cervical, ovarian) based on model’s Endometrial scores. The resulting confusion matrix on applying a threshold shows good accuracy, sensitivity and specificity. Panel (**B**) shows the results of specifically testing the multiclass trained model for separation of breast cancer samples from the other cancers (endometrial, cervical, and ovarian) based on model’s Breast scores. The resulting confusion matrix on applying a threshold shows good accuracy, sensitivity and specificity. Panel (**C**) the results of specifically testing the multiclass trained model for separation of cervical cancer samples from the other cancers (breast, endometrial, ovarian) based on model’s Cervical scores. The resulting confusion matrix on applying a threshold shows good accuracy, sensitivity and specificity. Panel (**D**) shows the results of specifically testing the multiclass trained model for separation of ovarian cancer samples from the other cancers (breast, endometrial, cervical) based on model’s Ovarian scores. The resulting confusion matrix on applying a threshold shows high accuracy, sensitivity and specificity.

To determine how well our multiclass model differentiates between the individual disease categories of the BECO group of samples, as well as from normal control, we plotted the scores obtained from multiclass model. As shown in Fig. 7A, we plotted the multiclass model Endometrial Score for Endometrial Cancer samples and set of Breast, Cervical and Ovarian (BCO) Cancer samples. The model scores are clearly seen to be different between Endometrial and BCO Cancer samples where on applying a threshold to differentiate between the two sets results in a confusion matrix as shown in Fig. 7A. Here, the normal samples were also included in the control group to get the sensitivity, specificity values for Endometrial cancer versus the rest of the groups. Sensitivity, Specificity and Accuracy were calculated from formulae given in “Methods”, which resulted in Sensitivity of 87%, Specificity of 93%, and an Accuracy of 91.6%.

We next plotted multiclass model taking Breast Cancer Scores for Breast Cancer Samples versus the scores from the combined set of Endometrial, Cervical and Ovarian (ECO) Cancer samples (Fig. 7B). The model scores are clearly seen to be different between Breast Cancer and ECO Cancer samples where on applying a threshold to differentiate between two sets results in a confusion matrix as shown (Fig. 7B). Here, the normal samples are also included in the control group to get the sensitivity, specificity values for Breast cancer versus the remaining groups. Sensitivity, Specificity and Accuracy were calculated from formulae given in “Methods”: AI modeling of the data. This results in Sensitivity of 93%, Specificity of 95%, and an Accuracy of 94.4%.

Figure 7C, shows a plot of the multiclass model where the Cervical Score for Cervical Cancer were compared with the scores from the combined set of Endometrial, Breast and Ovarian (EBO) Cancer samples. The model scores are clearly seen to be different between Cervical and EBO Cancer samples where on applying a threshold to differentiate between two sets results in a confusion matrix as shown. Here, the normal control samples were also added in the control group to get the sensitivity, specificity values for Cervical versus rest. Sensitivity, Specificity and Accuracy were calculated from formulae given in “Methods” as Sensitivity of 87%, Specificity of 90%, and an Accuracy of 87.6%.

Figure 7D shows a plot of the multiclass model taking the Ovarian Score for Ovarian Cancer Samples versus scores from the combined set of Endometrial, Breast and Cervical (EBC) Cancer samples. The model scores are clearly seen to be different between Ovarian Cancer and EBC Cancer samples where on applying a threshold to differentiate between two sets results in a confusion matrix as shown. Here, the normal samples are also added to the control group to get the sensitivity, specificity values for Ovarian versus rest. Sensitivity, Specificity and Accuracy were calculated from formulae given in “Methods”, which resulted in Sensitivity of 86%, Specificity of 93%, and an Accuracy of 92%.

### Feature ranking and literature validation of select features

Since our matrix features identify named metabolites from the HMDB database, the resulting model becomes explainable in terms of extracting mechanistic and other relevant insights related to the women-specific cancers. To enable this, we performed feature ranking, where the weights of the individual features from the model were first sorted. Following this, an adding of one feature at a time approach was used until the desired sensitivity–specificity was obtained. The top 100 metabolites obtained for both AI models, AI model—1 and AI model—2 are present in Supplementary Table S3.

Table 2 gives a list of twenty-five metabolites from the top 100 metabolites identified in AI model-1, which contribute to distinguishing the women-specific cancer (BECO) group from normal controls. It is evident that list comprises of diverse classes of metabolites that include lipids, nucleosides/nucleotides, amino acids and modified amino acids, acylcarnitines, steroids and dipeptides. While all of these metabolites have been implicated either in tumor growth or progression (Table 2), it is evident that they span a multiplicity of both anabolic and catabolic pathways (Table 3). This is consistent with emerging evidence that tumor cells have highly complex metabolic requirements, and that numerous pathways are required to complement glucose- and glutamine-dependent biomass production^38^. Representative metabolites that comprise the signature that helps to distinguish the individual cancers (i.e. breast, endometrial, cervical, or ovarian cancer) are listed in Supplementary Tables 4–7.

**Table 2:** List of select metabolites involved in the signature for distinguishing the BECO cancer group from normal controls.

S. no	Metabolite name	Involvement in cancer	*m/z*	RT (min)
1.	2-[(5Z,8Z,11Z,14Z,17Z)-eicosapentaenoyl]-sn-glycerol	One of the sixteen diagnostic metabolites that are able to identify early-stage ovarian cancer with high accuracy [1]	376.259	9.238
2.	Varanic acid	A bile acid that has been identified as a potential biomarker in the serum of ovarian cancer patients [2]	436.317	9.589
3.	Serotonin	A known growth factor for human tumor cells of different origins; implicated in cancer cell migration, metastatic dissemination, and tumor angiogenesis [3]	176.094	1.696
4.	Pyridoxamine 5'-phospate	A vitamin B6 phosphate. Vitamin B6 and its derivatives are inversely associated with cancer risk [4]	248.056	3.847
5.	L-Proline	Proline availability influences collagen synthesis and maturation and the acquisition of cancer cell plasticity and heterogeneity [5]	115.063	0.709
6.	Lewis X	Lewis X is a type II Lewis antigen, representing a fucosylated epitope that is overexpressed on the surface of cancer cells [6]	529.198	4.726
7.	17-beta-Estradiol	Plays a key role in breast cancer and regulates cancer/immune cell interactions in the tumor microenvironment [7]	272.176	7.829
8.	7-Ketodeoxycholic acid	It was identified as a potential biomarker during the metabolic profiling of serum in ovarian cancer patients [8]	406.27	10.176
9.	PAF C-18:1	Platelet activating factors are frequently induced in cancer cell through the action of Epidermal Growth Factor [9]	549.377	9.374
10.	Leukotriene F4	Leukotrienes play intricate roles in promoting tumor growth and metastasis through shaping the tumor microenvironment [10]	496.258	10.236
11.	Leukotriene D4	Leukotriene D4 plays an intricate roles in promoting tumor growth and metastasis through shaping the tumor microenvironment [11]	496.258	10.236
12.	N-Acetyl-DL-Histidine	Identified to correlate with colorectal cancer in an analysis of the fecal metabolome [12]	197.079	10.16
13.	6-Sulfatoxymelatonin	Present in the urine of women with breast cancer, although the question of whether they correlate with cancer risk remains uncertain [13]	328.071	3.215
14.	Androstenedione	Is associated with increased risk for endometrial cancer in postmenopausal women. They likely influence endometrial carcinogenesis via estrogen metabolism [14]	286.192	5.219
15.	Nisinic acid	While Nisinic acid itself has not been well studied, there is increasing evidence that PUFAs play a role in cancer risk and progression [15]	356.27	8.176
16.	Formiminoglutamic acid	An intermediate in the degradative metabolism of histidine, elevated levels of this metabolite have been found in urine of patients with neoplastic disease [16]	174.063	1.21
17.	Androsterone glucuronide	A conjugated steroid and, along with other conjugated steroids, has been implicated in risk of developing hormone-dependent breast cancer [17]	466.254	8.517
18.	3-Methoxytyramine	A prognostic biomarker that associates with high-risk disease and poor clinical outcome in neuroblastoma patients. Role in women-specific cancers yet unknown [18]	167.094	1.43
19.	Dipeptides: Pro-Tyr, Asp-Gln, Lys-Tyr, Lys-Arg	Dipeptides with either Ala, Asp, or Ile at the C-terminus, and dipeptides with Lys, Arg, Pro, and Tyr at the N-terminus were found to be overabundantly present in the liver of patients with Hepatocellular carcinoma [19]	278.125	3.961
20.	LysoPC(P-18:0)	Prospective case-cohort studies have revealed that higher levels of LysoPC(P-18:0) were consistently related to lower risks of breast, prostate, and colorectal cancer [20]	507.366	11.098
21.	Platelet-activating factor	PAF has been implicated in development, growth, and metastatic manifestations of cancer cells [21]	523.361	11.205
22.	5-Methylcytidine	Its constituent base, 5-methylcytosine is a sensitive marker of progress of the tumor formation induced by the oxidative damage reactions [22]	257.1	0.849
23.	1-Methylinosine	Modified nucleosides such as 1-methylinosine represent accurate tumor markers for clinical diagnosis of cancer [23]	282.095	3.562
24.	L-Glutamine	Glutamine is essential for tumor growth and host glutamine depletion is a hallmark of progressive tumor growth [24]	199.095	5.672
25.	O-heptadecanoylcarnitine	One of the acylcarnitines that are increased in cancer patients, and in those patients with higher cancer grades [25]	413.348	9.052

Literature references for the individual metabolites are listed in Supplementary Information. The mean m/z and retention time (RT) values for each metabolite are given.

**Table 3:** Diverse biological processes are influenced by the perturbed metabolites specific for the BECO cancer group.

Metabolic pathways
Nitrogen metabolism
Glutamine/glutamate metabolism
Aminoacyl-t-RNA biosynthesis
Arginine biosynthesis
Histidine metabolism
Steroid hormone biosynthesis
Ether lipid metabolism
Alanine, aspartate, and glutamate metabolism
Glyoxalate and dicarboxylate metabolism
Arachidonic acid metabolism
Glycerophospholipid metabolism
Arginine and proline metabolism
Pyrimidine metabolism
Tryptophan metabolism
Tyrosine metabolism
Purine metabolism

Metabolanalyst software (https://www.metaboanalyst.ca/) was used for extracting the list of biological processes associated with metabolites of interest. Briefly, the set of 25 metabolite names were added to the metaboanalyst software which cross referenced it to KEGG pathways database and HMDB database to get KEGG and HMDB ids respectively of input metabolites. Following this, the associated biological processes were extracted.

## Discussion

With the increasing burden of cancer mortality in women^39,40^, early detection to improve treatment outcomes has now become a priority. Unfortunately, however, reliable and accurate methods for early detection of most cancers are still not available. The problem is further exacerbated, particularly in middle- to low-income settings, by the relatively high costs of cancer screening, especially because current methods largely allow only for diagnosing one cancer at a time. As opposed to this, any method that can simultaneously detect multiple cancers at the early stage would find greater applicability simply because of the fewer number of diagnostic procedures that will need to be undertaken, and also the associated reduction in cost that it will entail. The utility of such a multi-cancer detection test would be further enhanced if it involved a non-invasive procedure, and if the test accuracy were to be high.

In the present report we describe an integrated method that can simultaneously detect Stage 0/I of all four of the women-specific cancers with high sensitivity and specificity. The cancers detected are breast, endometrial, cervical, and ovarian cancers. Our approach combined an untargeted UHPLC-MS/MS analysis of the serum metabolome, with the subsequent interrogation of the data using machine learning algorithms. A key aspect of our data analysis pipeline was the generation of a matrix wherein spectral features from the mass spectrometry profiles of the samples were translated into known metabolites identified using the HMDB database. A PLSDA plot revealed that the information content in the matrix was sufficient to clearly distinguish the cancer groups from the normal control group, and also achieve at least a reasonable degree of resolution between the individual cancer subsets. Consequently, this matrix provided the basis for developing an AI algorithm for early-stage cancer detection. For this, we employed a two-step strategy. In the first step we developed an algorithm (AI model—1) for distinguishing between the cancer samples and normal control samples. As our results show, we were indeed successful in identifying the BECO group of samples with high sensitivity and specificity. Subsequent to this, a second AI model (AI model—2) was developed in order to distinguish between the individual cancers of the BECO group. For this a one-versus-rest (OVR) classifier multiclass classification model was developed and as shown here, this model yielded a reasonably high accuracy in terms of identifying the identifying the tissue of origin of the cancer in samples of the BECO group. Efforts are currently underway to further improve the accuracy of AI model – 2.

Thus, our studies show that combining untargeted metabolomics with machine learning approaches for data analysis provides an attractive way forward for developing highly accurate multi-cancer detection approaches. Furthermore, especially noteworthy about our results is the high detection accuracy obtained for early-stage cancers, which is significantly superior to that of the other approaches being explored to date^42–45^. It will of interest to determine if the scope of this approach can be expanded to include simultaneous detection of additional cancers. In addition, a more extensive sampling of patients across a wider diversity of racial and ethnic groups would also help in determining the robustness of the approach.

## Methods

### Study design and sample collection

All samples used in this study were purchased from three separate commercial biobanks: Dx Biosamples (San Diego, CA), Reprocell USA Inc. (Beltsville, MD), and Fidelis Research AD (Sofia, Bulgaria). From these sources, we obtained serum samples that were derived from treatment-naïve women patients with Stage 0 or Stage 1 of either breast, uterine, cervical, or ovarian cancer. Clinical profile information on the donors included histological stage and grade, along with TNM classification of the cancer. Further, the HPV status of donors with uterine, cervical, and ovarian cancers was also provided; along with results of the CA-125 tumor marker determination for uterine and ovarian cancer patients. Finally, the breast cancer samples were also provided along with information on presence or absence of the Ki-67, ER, PR, and HER2 markers in the donors. To serve as controls in our assay, we also procured additional sera that were from normal volunteers. The total number of samples across all groups was 1369, and they were stored at − 20° C for the short term prior to use.

### Sample accessioning

Samples were inventoried and immediately stored at − 80 °C after receipt. Each sample received was allotted a unique identifier number. This identifier was used to trail all sample handling, tasks, results, etc. The samples (and all derived aliquots) were tracked by the identifier. All samples were maintained at − 80 °C until processed.

### Extraction of metabolites from serum samples

Metabolite extraction from serum was achieved as previously^34^. Briefly, all the serum samples were thawed on ice and mixed properly. 10 µl of each serum sample was taken in microfuge tube (1.5 ml), (Genaxy, Cat No. GEN-MT-150-C. S) and then 30 µl of chilled Methanol, (Merck, Cat.No.1.06018.1000) to the sample, vortexed briefly and then kept at − 20 ℃ for 60 min.

The sample was then centrifuged (Sorvall Legend Micro17, Thermo Fisher Scientific, Cat.No. Ligend Micro 17) at 10,000 rpm for 10 min. After centrifugation 27ul supernatant was collected in separate microfuge tube without disturbing the pellet and dried using Speed Vacuum, (ThermoFisher Scientific, Cat.No. SPD1030-230) at low energy for 30–35 min. Samples pellets were then re-suspended using 30 ul methanol: water (1:1, water: methanol) mixture for injection.

### Ultrahigh performance liquid chromatography-tandem mass spectroscopy (UHPLC-MS/MS)

Untargeted metabolomics were performed using Dionex LC system (Ultimate 3000) coupled online with QExactive Plus (Thermo Scientific). Each extracted metabolite sample was injected (10ul for positive ESI ionization) onto Acquity UPLC HSS T3 from Waters (1.8 micron, dimensions – 2.1 × 100 mm, Part No. 186003539), which was heated to 37^0^ C. The flow rate was 0.3 ml/min. Mobile phase A was (water + 0.1% formic acid), and mobile phase B was (methanol + 0.1% formic acid). The mobile phase was kept isocratic at 5% B for 1 min, and was increased to 95% B in 7 min and kept for another two min at 95% B, the mobile phase composition returned to 5% B in 14 min. The ESI voltage was 4 kV. The mass accuracy of QExactive mass spectrometry was less than 5 ppm and calibrated at recommended schedule prior to each batch run. The mass scan range is from 66.7 to 1000 Da, and resolution was set to 70,000. The maximum inject time for orbitrap was 100 ms while, AGC target was optimized with 1e6.

### Quality assessment and quality control

**Figure 8:**
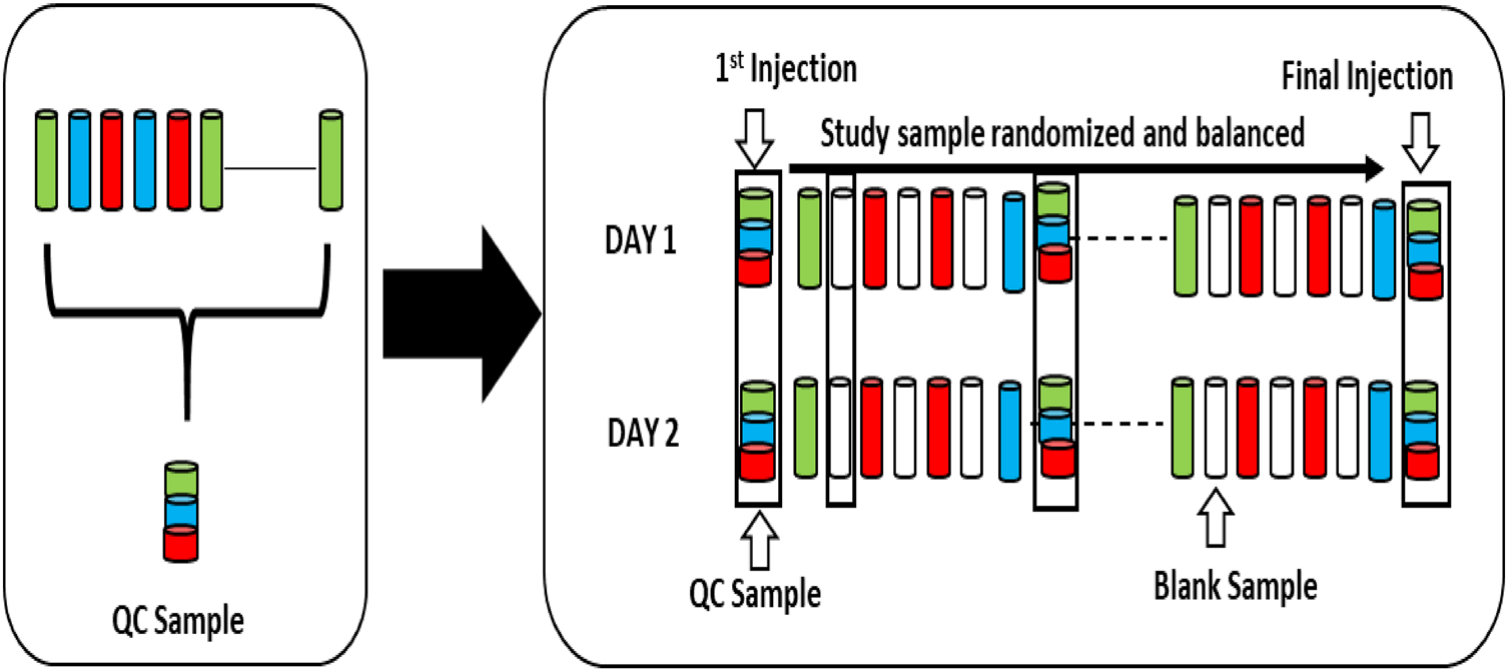
Preparation and scheduling of QC and samples for UHPLC-MS/MS. A small aliquot of each sample (coloured cylinders) was pooled to create a QC sample (multi-coloured cylinder), which was then injected periodically (every 50th injection) throughout the batch run. Variability among consistently detected metabolites was used to estimate overall process and batch variability. Every sample injection was followed by a blank injection to prevent carryover between the sample runs.

Several types of controls were analyzed in concert with the experimental samples: blank gradient runs were provisioned at every alternate sample run; a pooled QC sample generated by taking a small volume of each experimental sample, served as a technical replicate throughout the data set; also allowed instrument performance monitoring and aided chromatographic alignment. Mass accuracy of the instrument was checked on every 3rd day using the vendor specific calibrant (Thermo Fisher Scientific, Breda, The Netherlands). Overall process variability was determined by calculating the median RSD for all endogenous metabolites (i.e., non-instrument standards) present in 100% of the pooled matrix samples. Experimental samples were randomized across the platform run with QC samples spaced evenly (every 50th sample) among the injections (Fig. 8).

### Data processing

The mass spectrometry data was first subjected to preprocessing as shown schematically in Fig. 3. The individual steps were as follows:

#### Incorporating mass errors in the data

Mass errors are known to be present in metabolomics data^35^. This means that the same identified metabolite in different samples would have slightly different mass. This creates problems when intensity of same metabolite has to be compared across samples. This intensity comparison is required in the downstream AI based analysis. Usually, a fixed window size of mass is used to align the samples, but here, we have used a sophisticated approach of using a parts per million (ppm) error-based approach. Briefly, we have adapted the virtual lock mass (VLM) based approach^35^. This is based on the principle that mass errors are known to increase with mass^35^. This, approach was used and adapted according to our datasets. This was done by combining the traditional VLM based approach with metabolite identification from HMDB database. Specifically, the VLM boxes were defined using the masses of metabolites identified by HMDB database search across multiple samples. This resulted in an initial matrix of 6893 metabolites or features. From this, we next removed all features that corresponded to either plant products, or drug and their metabolites. The total number of features in the resultant matrix were reduced to 5558.

#### Metabolite ions filtering

The metabolite ions were filtered based on their frequency of presence in the individual samples. A 20% cutoff was used, wherein those metabolites that were present in less than 20% of the samples were excluded. This resulted in a final matrix size of 2764 features, which was then taken for our subsequent analysis.

#### Data normalization

Owing to the variations in the metabolic data across various conditions of the mass spectrometer, normalization methods are needed to minimize the variations in the data^36^. Different normalization methods were tried such as Quantile Normalization, Variance Stabilization Normalization, Best Normalization, Probabilistic Quotient Normalization. Quantile Normalization (QN) was selected as the one performing best across various conditions of the experiment. QN method was further adapted to our datasets to enable normalization of new samples with respect to training datasets and testing of one sample at a time.

#### Missing value imputation

Missing values in untargeted metabolomics data is known to be problematic^37^. A k-nearest neighbors (KNN) approach was applied to impute the missing values in the data to make the data more homogenous and amenable to AI based analysis.

#### AI modeling of the data

Now with the above data, AI models were made to differentiate cancers from normal and then between the individual cancers. Keeping in mind clinical applications of our AI model, a layered approach was used here in which first, an AI model was developed to differentiate BECO cancers from normal controls and then individual cancers. Briefly, logistic regression models were applied on the training data and tested on test data to give accuracy, sensitivity and specificity values according to formulae below:

**Table:** 

	Predicted		
Actual		Negative	Positive
	Negative	True negative (TN)	False positive (FP)
	Positive	False negative (FN)	True positive (TP)

$$\mathrm{Accuracy}: \frac{TP+TN}{TP+TN+FP+FN}$$

$$\mathrm{Sensitivity}:\frac{TP}{TP+FN}$$

$$\mathrm{Specificity}:\frac{TN}{TN+FP}$$

### Ethics statement

The study is in accordance with relevant guidelines and regulations.

## Supplementary Information

Supplementary Information.

Supplementary Table 3.

Supplementary Information.
