# Supplementary material for: A non-invasive method for concurrent detection of early-stage women-specific cancers
Source: Sci Rep. 2022 Feb 10;12:2301. doi: 10.1038/s41598-022-06274-9 (PMC8831619; doi:10.1038/s41598-022-06274-9)
Supplement: Supplementary file 1 — Supplementary Information. [file 41598_2022_6274_MOESM1_ESM.docx]

**A NON-INVASIVE METHOD FOR CONCURRENT DETECTION OF EARLY-STAGE WOMEN-SPECIFIC CANCERS**

**Ankur Gupta^1#^, Ganga Sagar^1#^, Zaved Siddiqui^1^, Kanury V.S. Rao^1,2^, Sujata Nayak^1,2^, Najmuddin Saquib^1*^ & Rajat Anand^1,2,*^**

**^1^PredOmix Technologies Private Limited**

**Tower B, SAS Tower**

**Medicity, Sector – 38**

**Gurugram – 122002,**

**India**

**^2^PredOmix, Inc.**

**9853 Pacific Heights Blvd.**

**San Diego, CA 92121-4721**

**USA**

^#^These authors contributed equally to this work.

^*^Address correspondence and requests for materials to RA (email: [rajat@predomix.com](mailto:rajat@predomix.com)) or NS (email: saquib@predomix.com)

**FIGURE LEGENDS**

**Figure S1. Distribution of the coefficient of each metabolite in the signature for the BECO group.**

See text for details

**Figure S2: Distribution of the coefficient of each metabolite in the signature for endometrial (A), breast (B), cervical (C), and ovarian (D) cancers.**

Details are provided in the text.

**REFERENCES**

1. **References for Table 2**

[1] Ref: Gaul, D.A., Mezencev, R., Long, T.Q., Jones, C. M., Bengino, B. B., Gray, A., Fernandez, F.M., & McDonald, J.F. Highly accurate metabolic detection of early-stage ovarian cancer. *Sci. Rep*. 2015, **5,** 16351 doi: [10.1038/srep16351](https://dx.doi.org/10.1038%2Fsrep16351) (2015).

[2] Ref. Yang, W., Mu, T., Jiang, J. Sun, Q, Hou, X., Sun, Y., Zhong, L., Wang, C., & Sun, C. Biomarkers and metabolic profiling of serum in ovarian cancer patients using UPLC/Q-TOF MS. *Cell. Physiol. Biochem*. **51**, 1134-1148 (2018).

[3] Ref: Sarrouilhe, D., & Mesnil, M. Serotonin and human cancer: A critical view. *Biochimie* **161,** 46-50 (2019).

[4] Ref: Mocellin, S., Briarava, M., & Pilati, P. Vitamin B6 and cancer risk: A field synopsis and meta-analysis. *J. Natl. Cancer Inst.* **109**, <https://doi.org/10.1093/jnci/djw230> (2017).

[5] Ref: D’Aniello, Patriarca, E. J., Phang, J., & Minchiotti, G. Proline metabolism in tumor growth and metastatic progression. *Front. Oncol.*  <https://doi.org/10.3389/fonc.2020.00776> (2020).

[6] Ref: Blanas, A., Sahasrabudhe, N. M., Rodriguez, E., van Kooyk, Y., % van Vljet, S. J. Front. Oncol. https://doi.org/10.3389/fonc.2018.00039 (2018)

[7] Ref: Rodriguez, G.V., Abrahamsson, A., Jensen, L. D. E., & Dabrosin, C. Estradiol promotes breast cancer cell migration via recruitment and activation of neutrophils. Cancer Immunol. Res. 5, 234-247 (2017)

[8] Ref. Yang, W., Mu, T., Jiang, J. Sun, Q, Hou, X., Sun, Y., Zhong, L., Wang, C., & Sun, C. Biomarkers and metabolic profiling of serum in ovarian cancer patients using UPLC/Q-TOF MS. Cell. Physiol. Biochem. 51, 1134-1148 (2018)

[9] Ref: Yu Y, Zhang X, Hong S, Zhang M, Cai Q, Jiang W, & Xu C. Epidermal growth factor induces platelet-activating factor production through receptors transactivation and cytosolic phospholipase A2 in ovarian cancer cells. J Ovarian Res. 2014;7:39. doi: 10.1186/1757-2215-7-39.

[10]Ref: Tian, W., Jiang, X., Kim, D., Guan, T., Nicolls, M. R., & Rockson, S. G. Leokotrienes in tumor-associated inflammation. Front. Pharmacol. https://doi.org/10.3389/fphar.2020.01289 (2020)

[11] Ref: Tian, W., Jiang, X., Kim, D., Guan, T., Nicolls, M. R., & Rockson, S. G. Leokotrienes in tumor-associated inflammation. Front. Pharmacol. https://doi.org/10.3389/fphar.2020.01289 (2020)

[12] Ref: Sinha R, Ahn J, Sampson JN, Shi J, Yu G, Xiong X, Hayes RB, Goedert JJ: Fecal Microbiota, Fecal Metabolome, and Colorectal Cancer Interrelations. PLoS One. 2016 Mar 25;11(3):e0152126. doi: 10.1371/journal.pone.0152126. eCollection 2016.

[13] Ref: Xu, J., Huang, L, & Sun, G-P. Urinary 6-sulfatoxymelatonin level and breast cancer risk: systematic review and meta-analysis. Sci. Rep. 7, doi: 10.1038/s41598-017-05752-9 (2017)

[14] Ref: Michels, K. A., Brinton, L. A., Wentzensen, N., Pan, K., Chen, C., Anderson, G. L., Pfeiffer, R., Xu, X., Rohan, T. E., & Trabert, B. Postmenopausal androgen metabolism and endometrial cancer risk in the Women’s Health Observational Study. JNCI Cancer Spectrum 3, pkz029, https://doi.org/10.1093/jncics/pkz029 (2019)

[15] Ref: Azrad, M., Turgeon, C., & Demark-Wahnefried, W. Current evidence linking polyunsaturated fatty acids with cancer risk and progression. Front. Oncol. 04 Sept. https://doi.org/10.3389/fonc.2013.00224 (2013)

[16] Ref: Carey, R. W., Brena, G. P., & Krant, M. J. Urinary Formiminoglutamic acid excretion in patients with neoplastic disease. Cancer 17, 713-722 (1964)

[17] Ref: McNamara, K. M., Nakamura, Y., Miki, Y., & Sasano, H. Phase two steroid metabolism and its roles in breast and prostate cancer patients. Front. Endocrinol. 04 Sept. https://doi.org/10.3389/fendo.2013.00116 (2013)

[18] Ref: Verly, R. N., van Kuilenburg, A. B. P., Abeling, N. G. G. M., Goorden, S. M. I., Fiocco, M., van Noesel, M. M., Zwaan, C. M. Z., Kaspers, G. J. L., Merks, J. H. M., Caron, H. N., & Tytgat, G. A. M. 3-Methoxytyramine: An independent prognostic biomarker that associates with high-risk disease and poor clinical outcome in neuroblastoma patients. Eur. J. Cancer 90, 102-110 (2018)

[19] Ref: Ozawa, H., Hirayama, A., Shoji, F., Maruyama, M., Suzuki, K., Yamanaka-Okumura, H., Tatano, H., Morine, Y., Soga, T. Shimada, & Tomita, M. Comprehensive dipeptide analysis revealed cancer-specific profile in liver of patients with hepatocellular carcinoma and hepatitis. Metabolites 10, doi:10.3390/metabo10110442 (2020)

[20] Ref: Kuhn, T., Floegel, A., Sookthal, D., Johnson, T., Rolle-Kampczyk, U., Otto, W., von Bergen, M., Boeing, H., & Kaaks, R. Higher plasma levels of lysophosphatidylcholine 18:0 are related to a lower risk of common cancers in a prospective metabolomics study

[21] Ref: Tsoupras, A. B., Iatrou, C., Frangia, C., & Demopoulos, C. A. The implication of platelet activating factor in cancer growth and metastasis: potent beneficial role of PAF-inhibitors and antioxidants. Infect. Disord Drug Targets 9, 390-399 (2009)

[22] Ref: Barciszewska, A., Murawa, D., Gawronska, I., Murawa, O., Nowak, S., & Barciszewska, M. Z. Analysis of 5-methylcytosine in DNA of breast and colon cancer tissues. IUBMB Life 59, 765-770 (2007)

[23] Ref: Seiudel, A., Brunner. P., Seidel, P., Fritz, G. I., & Herbarth, O. Modified nucleosides: an accurate tumor marker for diagnosis of cancer, early detection and therapy control. Br. J. Cancer 94, 1726-1733 (2006).

[24] Ref: Souba, W. W., Glutamine and cancer. Ann Surg 218, 715-728 (1993)

[25] Ref: Console, L., Scalise, M., Mazza, T., Pochini, L., Gallucio, M., Giangregorio, N., Tonazzi, A., & Indiveri, C. Carnitine traffic in cells: link with cancer. *Front. Cell Dev. Biol.* 18 Sept. <https://doi.org/10.3389/fcell.2020.583850> (2020)

1. **References for Table S4**

[1] Ref: Knott, S. R., Wagenblast, E., Khan, S., Kim, S. Y., Soto, M., et. al. Asparagine bioavailability governs metastasis in a model of breast cancer. Nature 554, 378-381 (2018)

[2] Ref: Roch, A. M., Noel, P., el Alaoul, S., & Quash, G. Differential expression of isopeptide bonds N epsilon (gamma-glutamyl)lysine in benign and malignant human breast lesions: an immunohistochemical study. Int. J. Cancer 48, 215-220 (1991)

[3] Ref: Krashin, E., Piekielka-Witkowska, A., Ellis, M., & Ashur-Fabian, O. Thyroid hormones and cancer: A comprehensive review of preclinical and clinical studies. Front. Endocrinol. 13th Feb. https://doi.org/10.3389/fendo.2019.00059 (2019)

[4] Ref: de La Puente-Yague, M., Cuadrado-Cenzual, M. A., Ciudad-Cabanas, M., Hernandez-Cabria, M., & Collado-Yurrita, L. Vitamin D: and its role in breast cancer. Kaohsiung J. Med. Sci. 34, doi: 10.1016/j.kjms.2018.03.004 (2018)

[5] Ref: Li, H., Lee, M-H., Liu, K., Wang, T., Song, M., Han, Y., Yao, K., Xie, H., Zhu, F., Grossmann, M., Cleary, M. P., Chen,. W., Bode, A. M., & Dong, Z. Inhibitng breast cancer by targeting the thromboxane A2 pathway. Npj Precision Onc 1, https://doi.org/10.1038/s41698-017-0011-4 (2017)

[6] Ref: Jones, M. K., Ramsay, I. D., & Collins, W. P. Concentration of testosterone glucuronide in urine from women with breast tumours. Br. J. Cancer. 35, 885-887 (1977)

[7] Ref: Moro, K., Kawaguchi, T., Tsuchida, J., Gabriel, E., Qi, Q., Yan, L., Wakai, T., Takabe, K., & Nagahashi, M. Ceramide species are elevated in human breast cancer and are associated with less aggressiveness. Oncotarget 9, 19874-19890 (2018)

[8] Ref: Kingsnorth, A. N., Wallace, H. M., Bundred, N. J., & Dixon, J. M. Polyamines in breast cancer. Br. J. Surg. 71, 352-356 (1984)

[9] Ref: Fichna, J., & Janecka, A. Opioid peptides in cancer. Cancer and Metastasis Rev. 23, 351-366 (2004)

[10] Ref: Costarelli, V., & Sanders, T. A. B. Plasma deoxycholic acid concentration is elevated in postmenopausal women with newly diagnosed breast cancer. Eur. J. Clin. Nutr. 56, 925-927 (2002).

1. **References for Table – S5**

[1] Ref: Tian, W., Jiang, X., Kim, D., Guan, T., Nicolls, M. R., & Rockson, S. G. Leokotrienes in tumor-associated inflammation. Front. Pharmacol. https://doi.org/10.3389/fphar.2020.01289 (2020)

[2] Ref: Thapa, M., & Dallmann, G. Role of coenzymes in cancer metabolism. Sem. Cell & Dev. Biol. 98, 44-53 (2020)

[3] Ref: Mittal, P., Briggs, M., Klinger-Hoffmann, M., Kaur, G., Packer, N. H., Oehler, M. K., & Hoffmann, P. Altered N-linked glycosylation in endometrial cancer. Anal. Bioanal. Chem. 413, 2721-2733 (2021)

[4] Ref: de La Puente-Yague, M., Cuadrado-Cenzual, M. A., Ciudad-Cabanas, M., Hernandez-Cabria, M., & Collado-Yurrita, L. Vitamin D: and its role in breast cancer. Kaohsiung J. Med. Sci. 34, doi: 10.1016/j.kjms.2018.03.004 (2018)

[5] Ref: Sarrouilhe, D., & Mesnil, M. Serotonin and human cancer: A critical view. Biochimie 161, 46-50 (2019)

[6] Ref: Knapp, P. K., Baranowski, M., Knapp, M., Zabielski, P., Blachino-Zabielska, A. U., & Gorski, J. Altered sphingolipid metabolism in human endometrial cancer. Prostaglandins Other Lipid Mediat. 92, 62 – 66 (2010)

[7] Ref: Smith, P. G., Roque, D., Ching, M., Fulton, A., Rao, G., & Reader, J. C. The role of eicosanoids in gynecological malignancies. Front. Pharmacol. 11, doi: 10.3389/fphar.2020.01233 (2020)

[8] Ref: Tokarz, J., Adamski, J., & Rizner, T. L. Metabolomics for diagnosis and prognosis of uterine disease? A systematic review. J. Pers. Med. 10, doi: 10.3390/jpm10040294 (2020)

[9] Ref: Jara-Gutierrez, A., & Baladron, V. The role of prostaglandins in different types of cancer. Cells 10, 1487. https://doi.org/10.3390/cells10061487 (2021)

[10] Ref: Cruz-Lopez, K G., Castro-Munoz, L. J., Reyes-Hernandez, D. O., Garcia-Carranca, A., & Manzo-Merino, J. Lactate in the regulation of tumor microenvironment and therapeutic approaches. Front. Oncol. Nov. https://doi.org/10.3389/fonc.2019.01143 (2019).

1. **References for Table S-6**

[1] Ref: Tian, W., Jiang, X., Kim, D., Guan, T., Nicolls, M. R., & Rockson, S. G. Leokotrienes in tumor-associated inflammation. Front. Pharmacol. https://doi.org/10.3389/fphar.2020.01289 (2020)

[2] Ref: Melone, M. A., B., Velentino, A., Margarucci, S., Galderisi, U., Giordano, A., & Peluso, G. The carnitine system and cancer metabolic plasticity. Cell Death Dis. 9, doi: 10.1038/s41419-018-0313-7 (2018)

[3] Ref: Sharma, B., & Kanwar, S. S. Phosphatidylserine: a cancer cell targeting biomarker. Semin. Cancer Biol. 52, 17-25 (2018)

[4] Ref: Wang, Q., Gao, P., Wang, X., & Duan, Y. The early diagnosis and monitoring of squamous cell carcinoma via saliva metabolomics. Sci. Rep. 4, 6802, https://doi.org/10.1038/srep06802 (2014)

[5] Ref: Zeleniuch-Jacquotte, A., Shore, R. E., Afanasyeva, Y., Lukanova, A., Sieri, S., Koenig, K. L., Idahl, A. Krogh, V., Liu, M., Ohlson, N., Muti, P., Arslan, A. A., Lenner, P., Berrino, F., Hallmans, G., Toniolo, P., & Lundin, E. Postmenopausal circulating levels of 2- and 16alpha-hydroxyestrone and risk of endometrial cancer. Br. J. Cancer 105, 1458-1464 (2011)

[6] Ref: Boutra, S., Aziat, F., Mandal, R., Guo, A. C. Wilson, M. R. et. al. The human urine metabolome. PLoS One 8, e73076.

doi: 10.1371/journal.pone.0073076. (2013)

[7] Ref: Ho, P-C., Bihuniak, J. D., Macintyre, A. N., Staron, M., Liu, X., et. al. Phosphoenolpyruvate is a metabolic checkpoint of anti-tumor T cell responses. Cell 162, https://doi.org/10/1016/j.cell.2015.08.012 (2015)

[8] Ref: Kiguchi, K., Iwamori, M., Yamanouchi, S., Ishiwata, I., Saga, M., & Amemiya, A. Coexpression of cholesterol sulfate and cytokeratin as timor markers in well-differentiated squamous cell carcinoma of the human uterine cervix. Clin. Cancer Res. 4, 2985-2990 (1998)

[9] Ref: Nuranna, L., Nuryanto, K. H., Andriansyah, A., Elvira, S. D., & Sutrisna, B. Changes in cortisol levels before and after supportive psychotherapy in patients with comorbid cervical cancer distress with depression type. Indonesian J. Obst. & Gynecol. 6, https://doi.org/10.32771/inajog.v6i3.784 (2018)

[10] Ref: Kiguchi, K., Iwamori, M., Yamanouchi, S., Ishiwata, I., Saga, M., & Amemiya, A. Coexpression of cholesterol sulfate and cytokeratin as timor markers in well-differentiated squamous cell carcinoma of the human uterine cervix. Clin. Cancer Res. 4, 2985-2990 (1998)

1. **References for Table S-7**

[1] Ref: Yusuke Kobayashi, Hiroyasu Kashima, Yohan Suryo Rahmanto, Kouji Banno, Yu Yu, Yusuke Matoba, Keiko Watanabe, Moito Iijima, Takashi Takeda, Haruko Kunitomi, Miho Iida, Masataka Adachi, Kanako Nakamura, Kosuke Tsuji, Kenta Masuda, Hiroyuki Nomura, Eiichiro Tominaga, and Daisuke Aoki, Drug repositioning of mevalonate pathway inhibitors as antitumor agents for ovarian cancer. Oncotarget. 2017 Sep 22; 8(42): 72147–72156. doi: 10.18632/oncotarget.20046

[2] Ref: P K Heinonen, T Koivula, P Pystynen. Decreased serum level of dehydroepiandrosterone sulfate in postmenopausal women with ovarian cancer. Gynecol Obstet Invest . 1987;23(4):271-4. doi: 10.1159/000298872.

[3] Hao Huang, Tian-Tian Tong, Lee-Fong Yau, Cheng-Yu Chen, Jia-Ning Mi, Jing-Rong Wang, and Zhi-Hong Jiang. LC-MS Based Sphingolipidomic Study on A2780 Human Ovarian Cancer Cell Line and its Taxol-resistant Strain. Sci Rep. 2016; 6: 34684. doi: 10.1038/srep34684

[4] Ref: Jennifer Ose, Elizabeth M. Poole, Helena Schock, Matti Lehtinen, Alan A. Arslan, Anne Zeleniuch-Jacquotte, Kala Visvanathan, Kathy Helzlsouer, Julie E. Buring, I-Min Lee, Anne Tjønneland, Laure Dossus, Antonia Trichopoulou, Giovanna Masala, N. Charlotte Onland-Moret, Elisabete Weiderpass, Eric J. Duell, Annika Idahl, Ruth C. Travis, Sabina Rinaldi, Melissa A. Merritt, Britton Trabert, Nicolas Wentzensen, Shelley S. Tworoger, Rudolf Kaaks, and Renée T. Fortner. Cancer Res. 2017 Jul 15; 77(14): 3951–3960. doi: 10.1158/0008-5472.CAN-16-3322

[5] Ref: Philip L Lorenzi 1, John N Weinstein. Asparagine synthetase: a new potential biomarker in ovarian cancer. Drug News Perspect . Jan-Feb 2009;22(1):61-4. doi: 10.1358/dnp.2009.22.1.1303820.

[6] Ref: Qiu, X., Cheng, J-C., Chang, H-M., & Leung, P. C. K. Cox2 and PGE2 mediate EGF-induced E-cadherin independent human ovarian cancer cell invasion. Endocr. Relat. Cancer 21, 533-543 (2014)

[7] Ref: Chaofu Ke 1, Yan Hou, Haiyu Zhang, Lijun Fan, Tingting Ge, Bing Guo, Fan Zhang, Kai Yang, Jingtao Wang, Ge Lou, Kang Li. Large-scale profiling of metabolic dysregulation in ovarian cancer. Int J Cancer 2015 Feb 1;136(3):516-26. doi: 10.1002/ijc.29010. Epub 2014 Jun 17.

[8] Ref: Sofia C. Nunes, Cristiano Ramos, Filipa Lopes-Coelho, Catarina O. Sequeira, Fernanda Silva, Sofia Gouveia-Fernandes, Armanda Rodrigues, António Guimarães, Margarida Silveira, Sofia Abreu, Vítor E. Santo, Catarina Brito, Ana Félix, Sofia A. Pereira & Jacinta Serpa. Cysteine allows ovarian cancer cells to adapt to hypoxia and to escape from carboplatin cytotoxicity. Scientific Reports volume 8, Article number: 9513 (2018). Doi: <https://doi.org/10.1038/s41598-018-27753-y>.

[9] Ref: Liting Sun,* Lu Chen,* Yanwen Jiang, Yun Zhao, Fengge Wang, Xue Zheng, Chunjin Li, and Xu Zhou. Biosci Rep. 2018 Dec 21; 38(6): BSR20180965. doi: 10.1042/BSR20180965

[10] Ref: Ho, P-C., Bihuniak, J. D., Macintyre, A. N., Staron, M., Liu, X., et. al. Phosphoenolpyruvate is a metabolic checkpoint of anti-tumor T cell responses. Cell 162, https://doi.org/10/1016/j.cell.2015.08.012 (2015)
