# Supplementary material for: A non-invasive method for concurrent detection of early-stage women-specific cancers
Source: Sci Rep. 2022 Feb 10;12:2301. doi: 10.1038/s41598-022-06274-9 (PMC8831619; doi:10.1038/s41598-022-06274-9)
Supplement: Supplementary file 2 — Supplementary Table 3. [file 41598_2022_6274_MOESM2_ESM.pptx]

## Slide 1
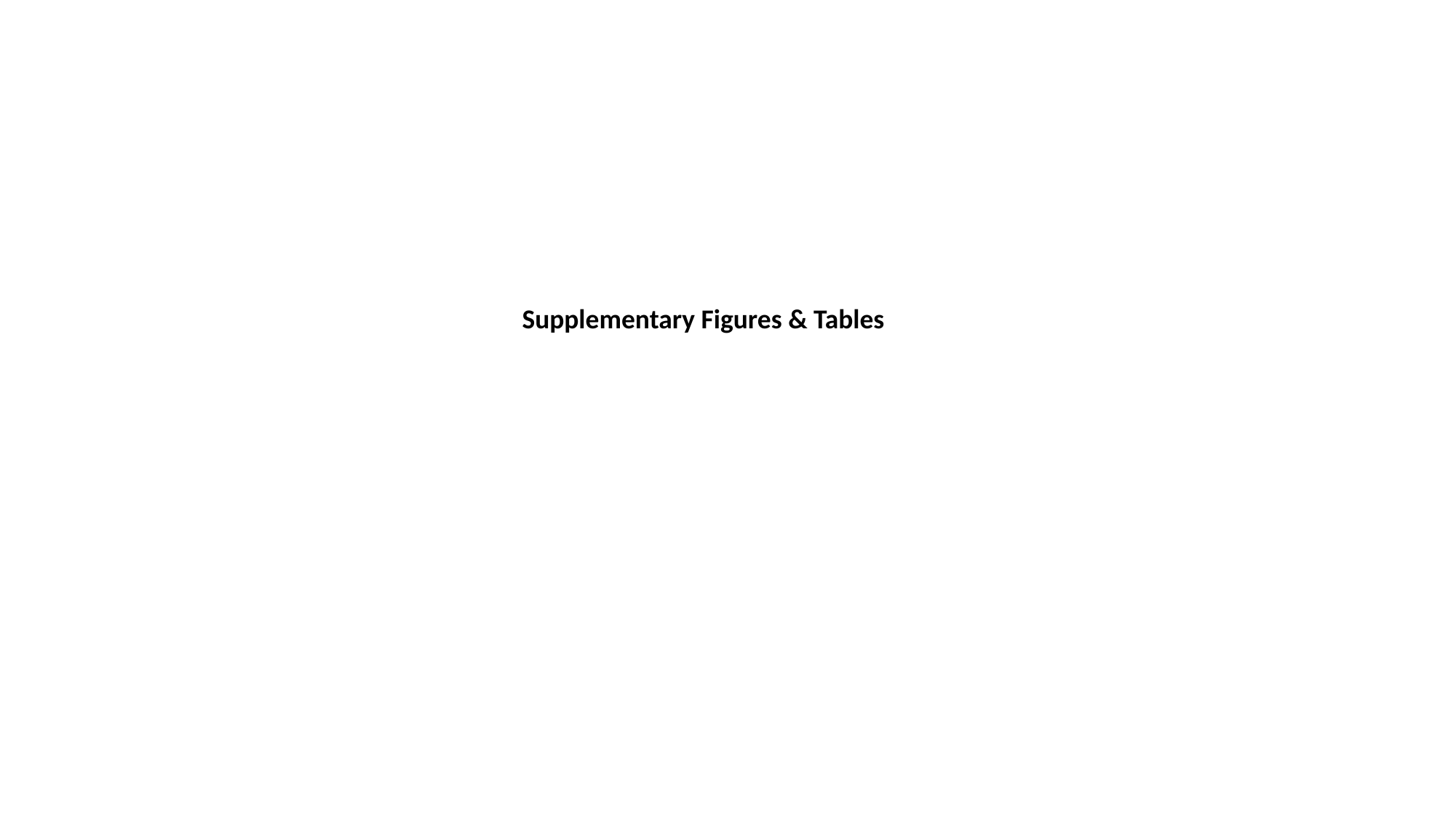

Supplementary Figures & Tables

## Slide 2
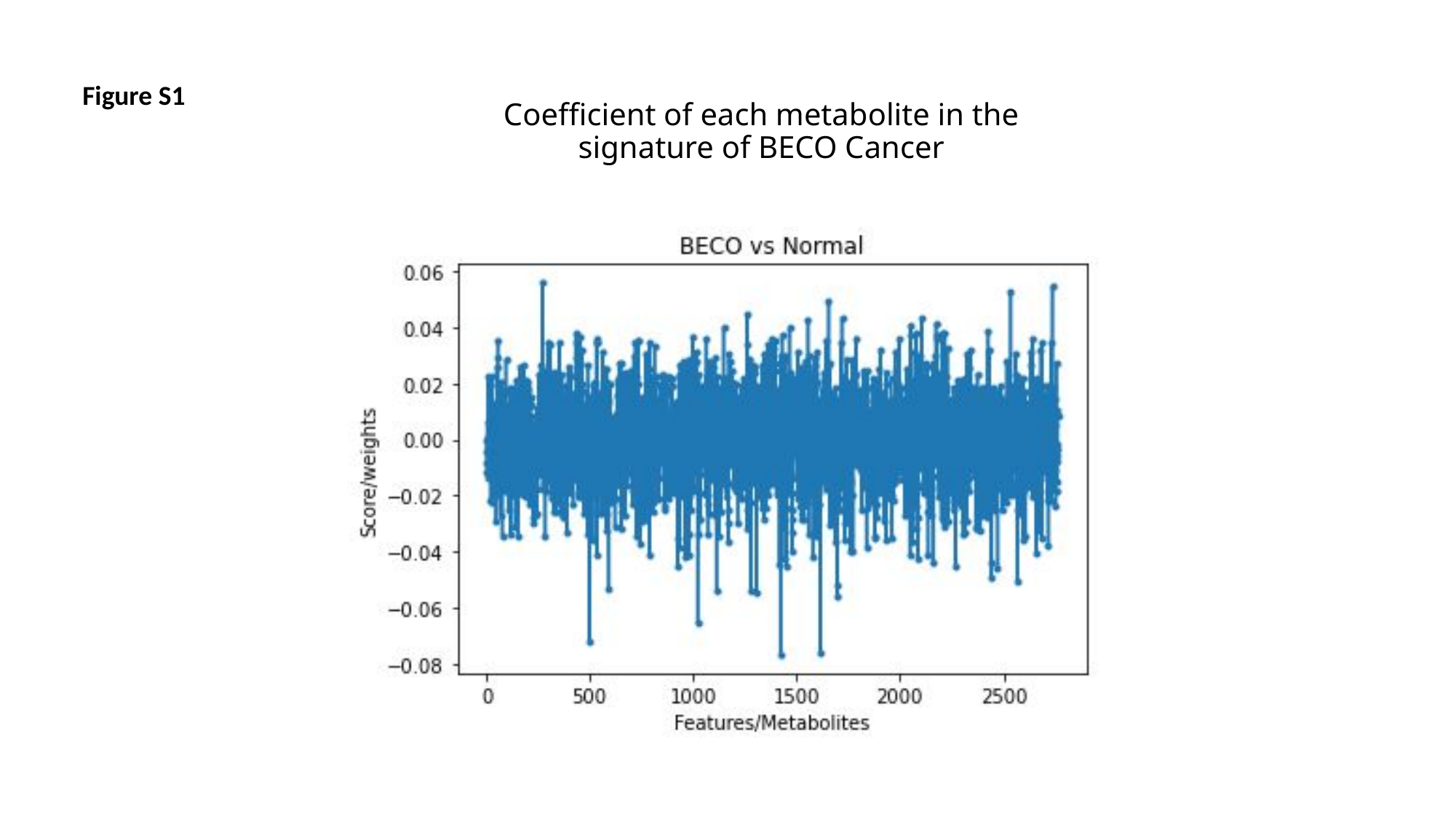

Figure S1
Coefficient of each metabolite in the signature of BECO Cancer

## Slide 3
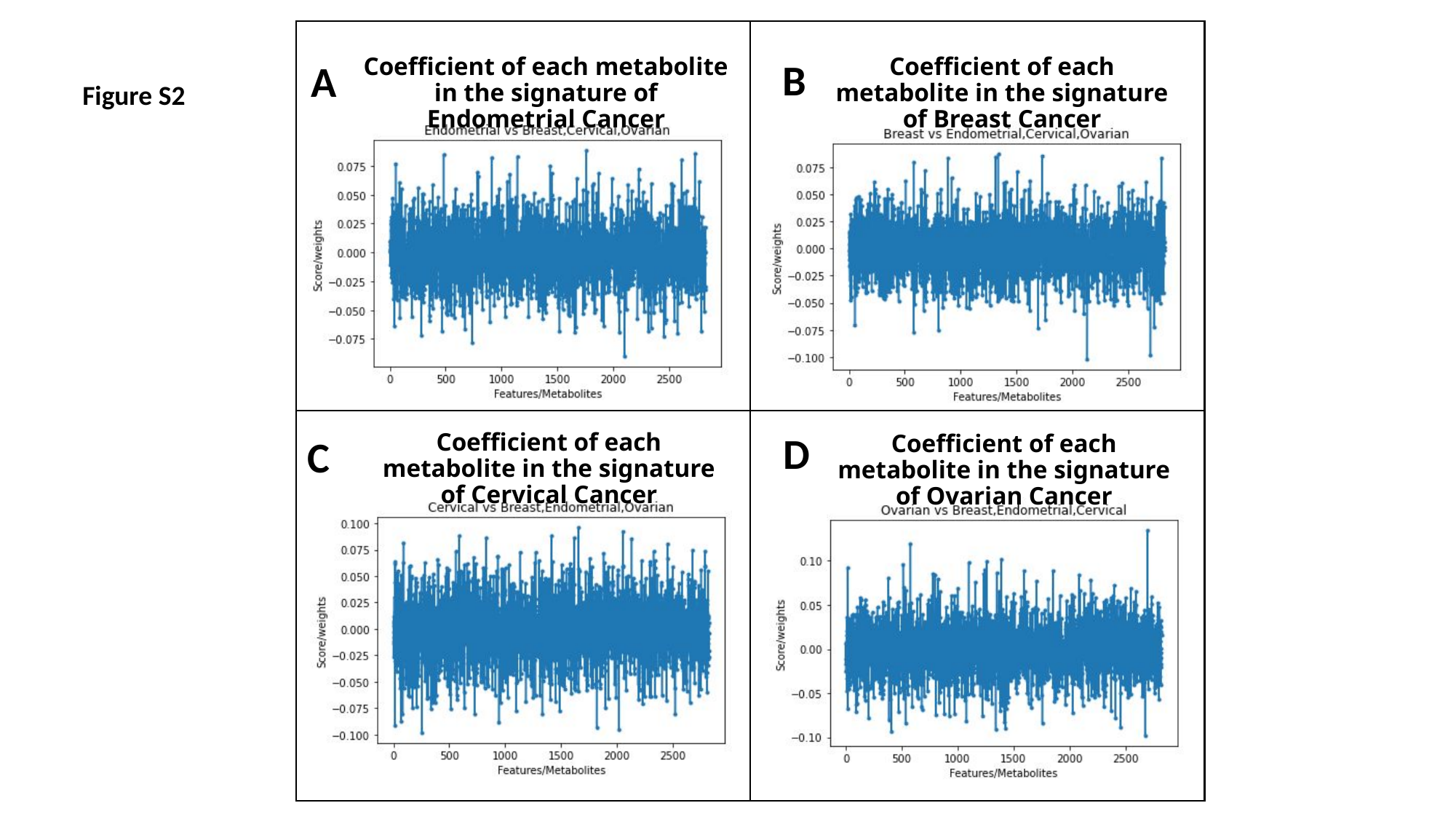

B
A
Coefficient of each metabolite in the signature of Endometrial Cancer
Coefficient of each metabolite in the signature of Breast Cancer
Figure S2
D
C
Coefficient of each metabolite in the signature of Cervical Cancer
Coefficient of each metabolite in the signature of Ovarian Cancer

## Slide 4
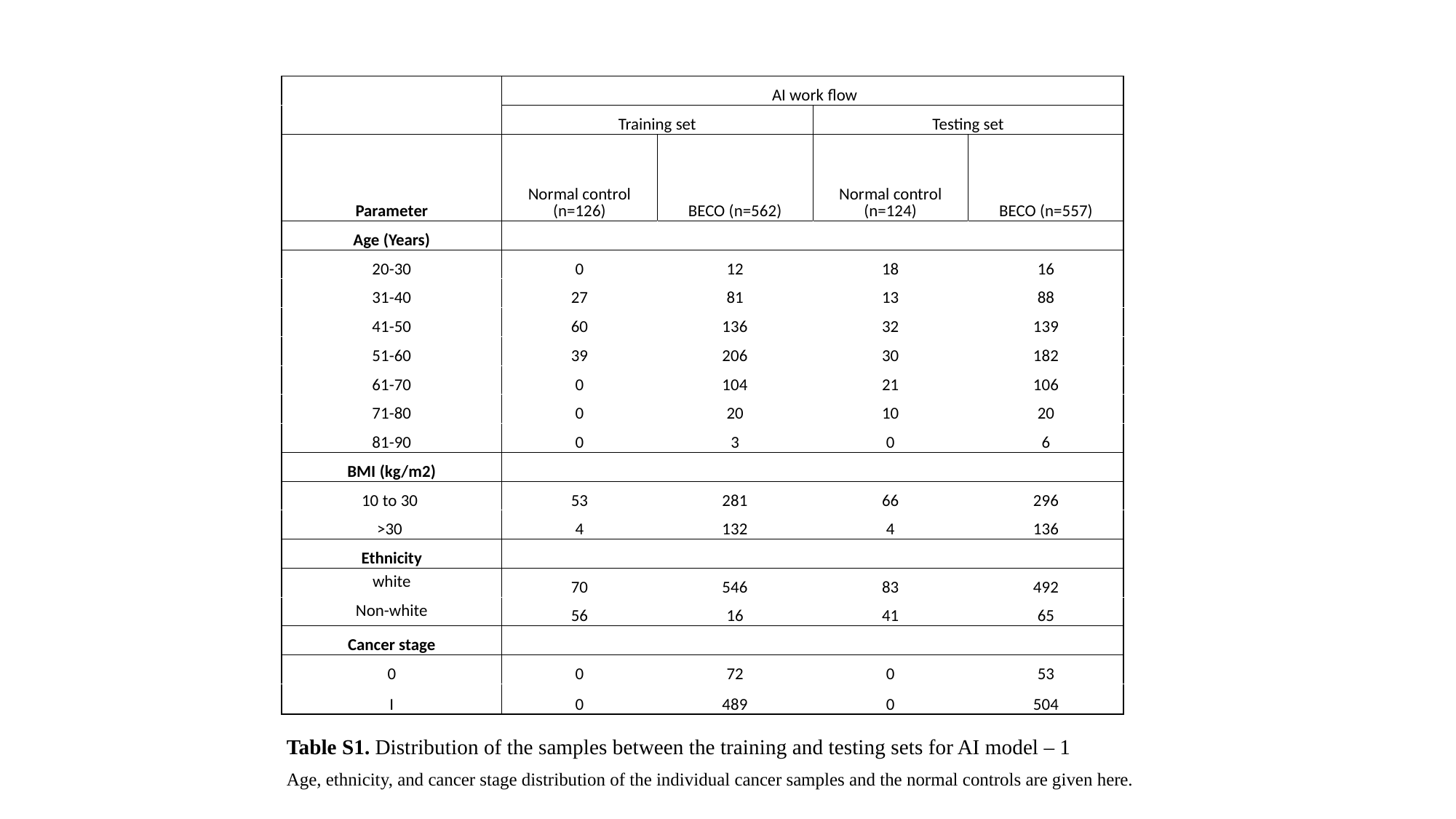

| | AI work flow | | | |
| --- | --- | --- | --- | --- |
| | Training set | | Testing set | |
| Parameter | Normal control (n=126) | BECO (n=562) | Normal control (n=124) | BECO (n=557) |
| Age (Years) | | | | |
| 20-30 | 0 | 12 | 18 | 16 |
| 31-40 | 27 | 81 | 13 | 88 |
| 41-50 | 60 | 136 | 32 | 139 |
| 51-60 | 39 | 206 | 30 | 182 |
| 61-70 | 0 | 104 | 21 | 106 |
| 71-80 | 0 | 20 | 10 | 20 |
| 81-90 | 0 | 3 | 0 | 6 |
| BMI (kg/m2) | | | | |
| 10 to 30 | 53 | 281 | 66 | 296 |
| >30 | 4 | 132 | 4 | 136 |
| Ethnicity | | | | |
| white | 70 | 546 | 83 | 492 |
| Non-white | 56 | 16 | 41 | 65 |
| Cancer stage | | | | |
| 0 | 0 | 72 | 0 | 53 |
| I | 0 | 489 | 0 | 504 |
Table S1. Distribution of the samples between the training and testing sets for AI model – 1
Age, ethnicity, and cancer stage distribution of the individual cancer samples and the normal controls are given here.

## Slide 5
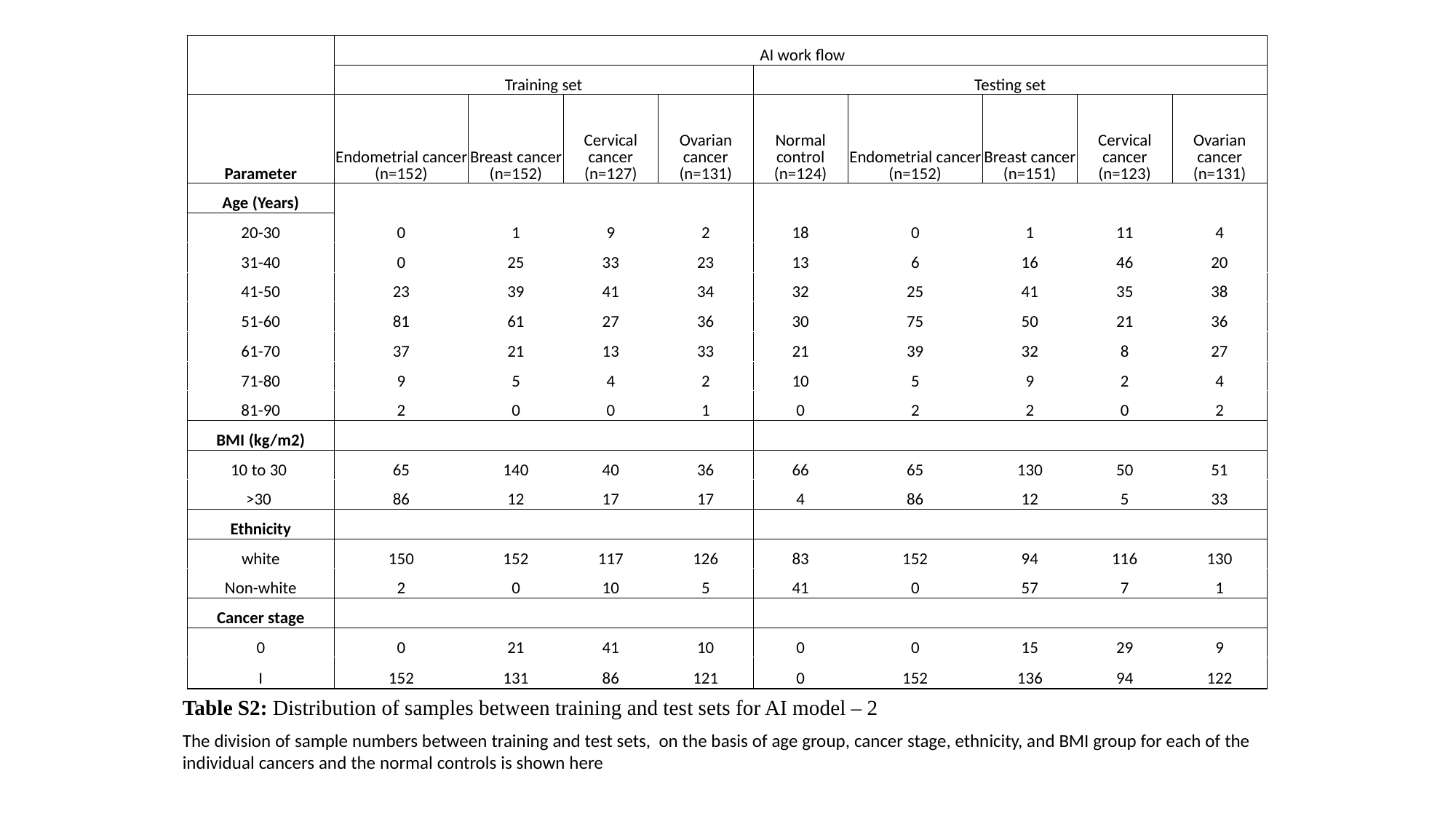

| | AI work flow | | | | | | | | |
| --- | --- | --- | --- | --- | --- | --- | --- | --- | --- |
| | Training set | | | | Testing set | | | | |
| Parameter | Endometrial cancer (n=152) | Breast cancer (n=152) | Cervical cancer (n=127) | Ovarian cancer (n=131) | Normal control (n=124) | Endometrial cancer (n=152) | Breast cancer (n=151) | Cervical cancer (n=123) | Ovarian cancer (n=131) |
| Age (Years) | | | | | | | | | |
| 20-30 | 0 | 1 | 9 | 2 | 18 | 0 | 1 | 11 | 4 |
| 31-40 | 0 | 25 | 33 | 23 | 13 | 6 | 16 | 46 | 20 |
| 41-50 | 23 | 39 | 41 | 34 | 32 | 25 | 41 | 35 | 38 |
| 51-60 | 81 | 61 | 27 | 36 | 30 | 75 | 50 | 21 | 36 |
| 61-70 | 37 | 21 | 13 | 33 | 21 | 39 | 32 | 8 | 27 |
| 71-80 | 9 | 5 | 4 | 2 | 10 | 5 | 9 | 2 | 4 |
| 81-90 | 2 | 0 | 0 | 1 | 0 | 2 | 2 | 0 | 2 |
| BMI (kg/m2) | | | | | | | | | |
| 10 to 30 | 65 | 140 | 40 | 36 | 66 | 65 | 130 | 50 | 51 |
| >30 | 86 | 12 | 17 | 17 | 4 | 86 | 12 | 5 | 33 |
| Ethnicity | | | | | | | | | |
| white | 150 | 152 | 117 | 126 | 83 | 152 | 94 | 116 | 130 |
| Non-white | 2 | 0 | 10 | 5 | 41 | 0 | 57 | 7 | 1 |
| Cancer stage | | | | | | | | | |
| 0 | 0 | 21 | 41 | 10 | 0 | 0 | 15 | 29 | 9 |
| I | 152 | 131 | 86 | 121 | 0 | 152 | 136 | 94 | 122 |
Table S2: Distribution of samples between training and test sets for AI model – 2
The division of sample numbers between training and test sets, on the basis of age group, cancer stage, ethnicity, and BMI group for each of the individual cancers and the normal controls is shown here

## Slide 6
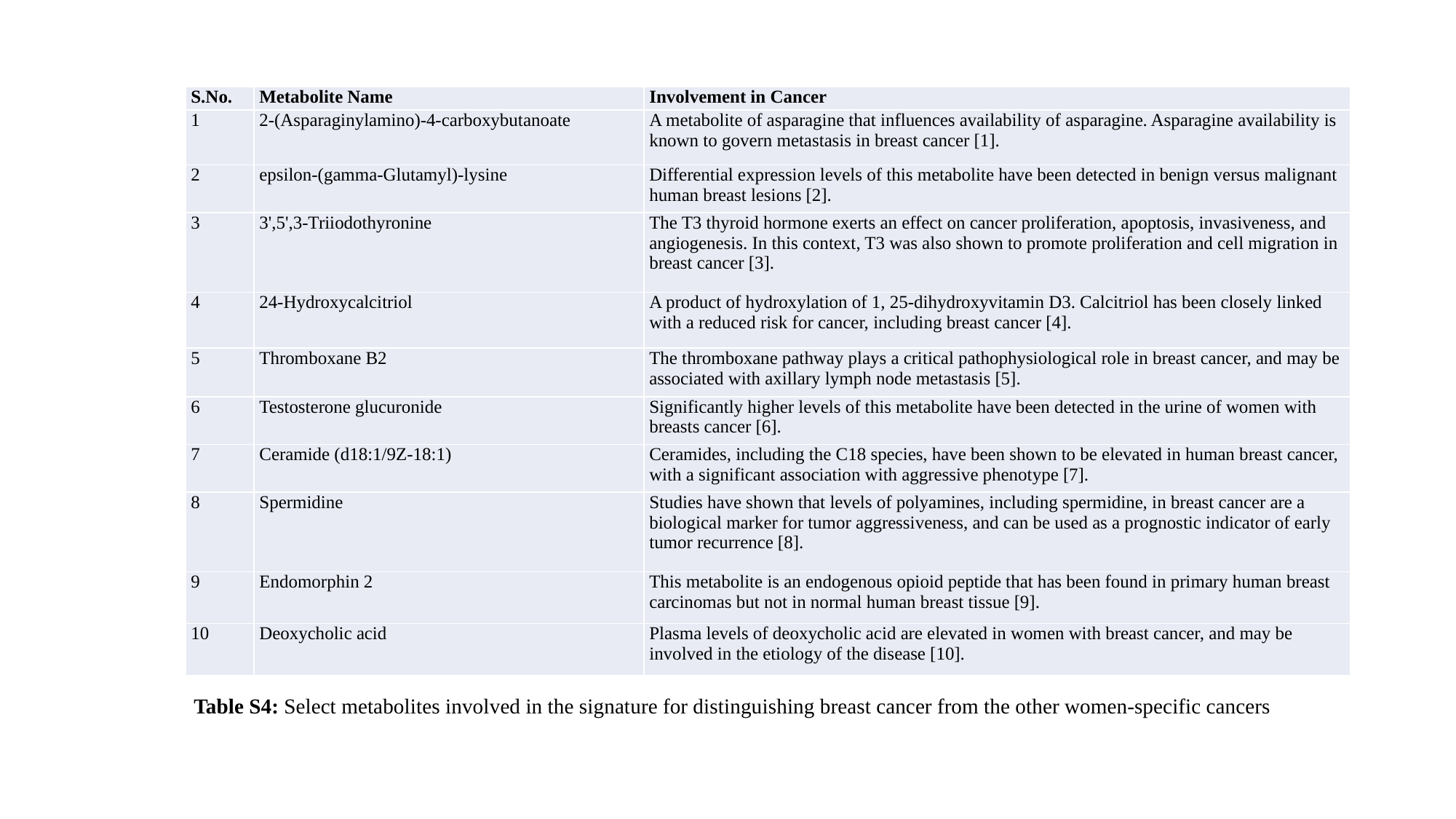

| S.No. | Metabolite Name | Involvement in Cancer |
| --- | --- | --- |
| 1 | 2-(Asparaginylamino)-4-carboxybutanoate | A metabolite of asparagine that influences availability of asparagine. Asparagine availability is known to govern metastasis in breast cancer [1]. |
| 2 | epsilon-(gamma-Glutamyl)-lysine | Differential expression levels of this metabolite have been detected in benign versus malignant human breast lesions [2]. |
| 3 | 3',5',3-Triiodothyronine | The T3 thyroid hormone exerts an effect on cancer proliferation, apoptosis, invasiveness, and angiogenesis. In this context, T3 was also shown to promote proliferation and cell migration in breast cancer [3]. |
| 4 | 24-Hydroxycalcitriol | A product of hydroxylation of 1, 25-dihydroxyvitamin D3. Calcitriol has been closely linked with a reduced risk for cancer, including breast cancer [4]. |
| 5 | Thromboxane B2 | The thromboxane pathway plays a critical pathophysiological role in breast cancer, and may be associated with axillary lymph node metastasis [5]. |
| 6 | Testosterone glucuronide | Significantly higher levels of this metabolite have been detected in the urine of women with breasts cancer [6]. |
| 7 | Ceramide (d18:1/9Z-18:1) | Ceramides, including the C18 species, have been shown to be elevated in human breast cancer, with a significant association with aggressive phenotype [7]. |
| 8 | Spermidine | Studies have shown that levels of polyamines, including spermidine, in breast cancer are a biological marker for tumor aggressiveness, and can be used as a prognostic indicator of early tumor recurrence [8]. |
| 9 | Endomorphin 2 | This metabolite is an endogenous opioid peptide that has been found in primary human breast carcinomas but not in normal human breast tissue [9]. |
| 10 | Deoxycholic acid | Plasma levels of deoxycholic acid are elevated in women with breast cancer, and may be involved in the etiology of the disease [10]. |
Table S4: Select metabolites involved in the signature for distinguishing breast cancer from the other women-specific cancers

## Slide 7
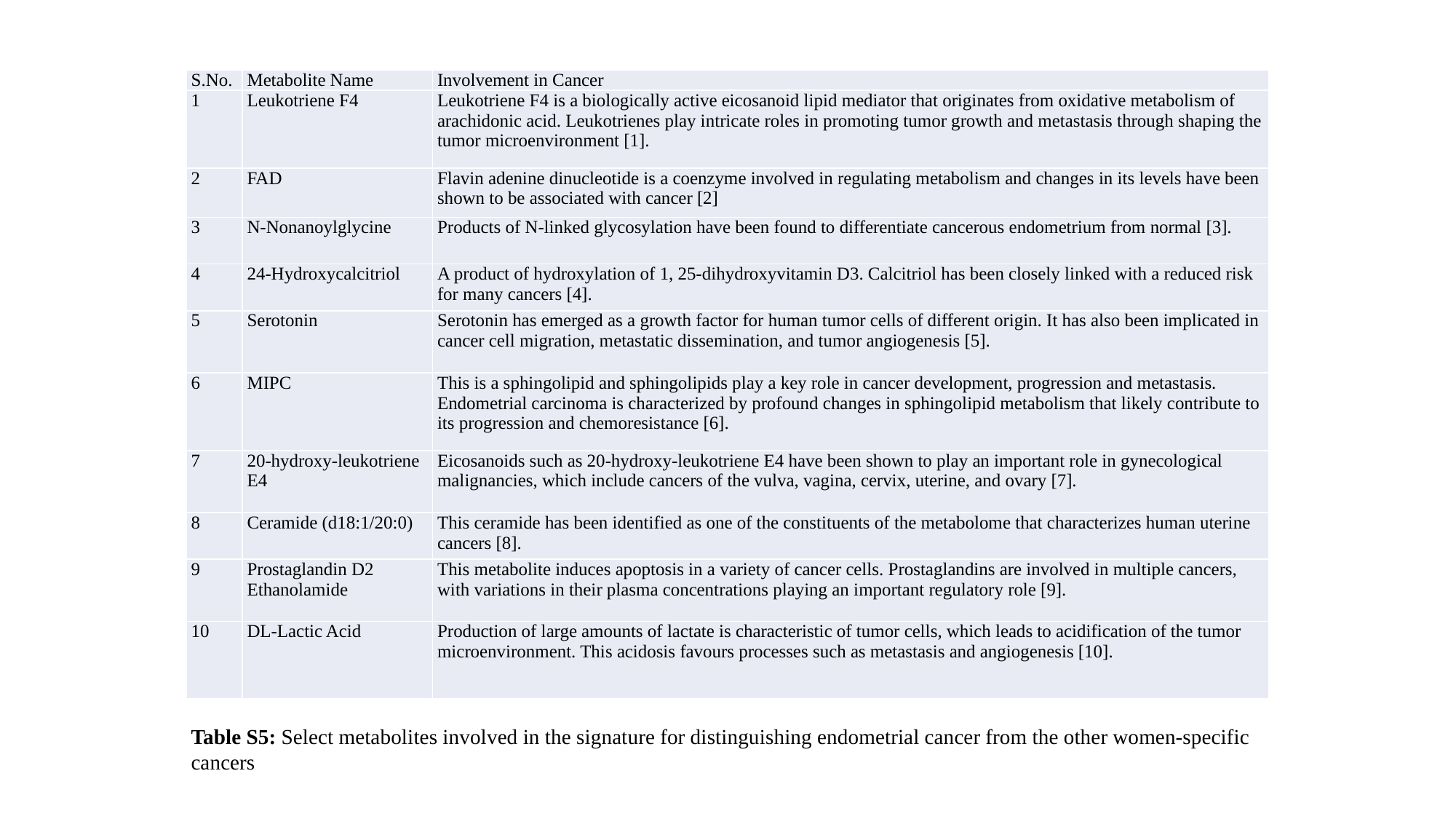

| S.No. | Metabolite Name | Involvement in Cancer |
| --- | --- | --- |
| 1 | Leukotriene F4 | Leukotriene F4 is a biologically active eicosanoid lipid mediator that originates from oxidative metabolism of arachidonic acid. Leukotrienes play intricate roles in promoting tumor growth and metastasis through shaping the tumor microenvironment [1]. |
| 2 | FAD | Flavin adenine dinucleotide is a coenzyme involved in regulating metabolism and changes in its levels have been shown to be associated with cancer [2] |
| 3 | N-Nonanoylglycine | Products of N-linked glycosylation have been found to differentiate cancerous endometrium from normal [3]. |
| 4 | 24-Hydroxycalcitriol | A product of hydroxylation of 1, 25-dihydroxyvitamin D3. Calcitriol has been closely linked with a reduced risk for many cancers [4]. |
| 5 | Serotonin | Serotonin has emerged as a growth factor for human tumor cells of different origin. It has also been implicated in cancer cell migration, metastatic dissemination, and tumor angiogenesis [5]. |
| 6 | MIPC | This is a sphingolipid and sphingolipids play a key role in cancer development, progression and metastasis. Endometrial carcinoma is characterized by profound changes in sphingolipid metabolism that likely contribute to its progression and chemoresistance [6]. |
| 7 | 20-hydroxy-leukotriene E4 | Eicosanoids such as 20-hydroxy-leukotriene E4 have been shown to play an important role in gynecological malignancies, which include cancers of the vulva, vagina, cervix, uterine, and ovary [7]. |
| 8 | Ceramide (d18:1/20:0) | This ceramide has been identified as one of the constituents of the metabolome that characterizes human uterine cancers [8]. |
| 9 | Prostaglandin D2 Ethanolamide | This metabolite induces apoptosis in a variety of cancer cells. Prostaglandins are involved in multiple cancers, with variations in their plasma concentrations playing an important regulatory role [9]. |
| 10 | DL-Lactic Acid | Production of large amounts of lactate is characteristic of tumor cells, which leads to acidification of the tumor microenvironment. This acidosis favours processes such as metastasis and angiogenesis [10]. |
Table S5: Select metabolites involved in the signature for distinguishing endometrial cancer from the other women-specific cancers

## Slide 8
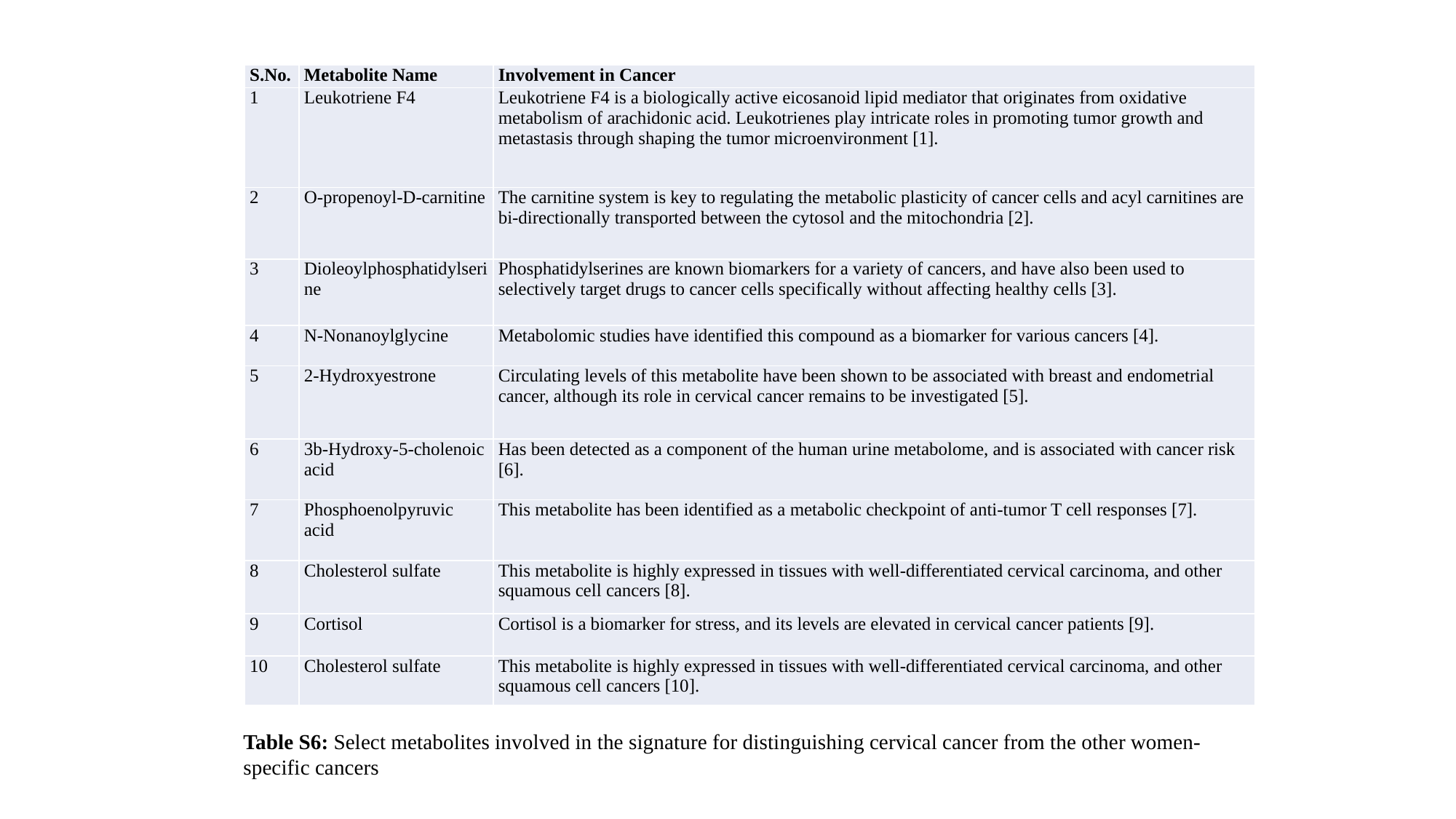

| S.No. | Metabolite Name | Involvement in Cancer |
| --- | --- | --- |
| 1 | Leukotriene F4 | Leukotriene F4 is a biologically active eicosanoid lipid mediator that originates from oxidative metabolism of arachidonic acid. Leukotrienes play intricate roles in promoting tumor growth and metastasis through shaping the tumor microenvironment [1]. |
| 2 | O-propenoyl-D-carnitine | The carnitine system is key to regulating the metabolic plasticity of cancer cells and acyl carnitines are bi-directionally transported between the cytosol and the mitochondria [2]. |
| 3 | Dioleoylphosphatidylserine | Phosphatidylserines are known biomarkers for a variety of cancers, and have also been used to selectively target drugs to cancer cells specifically without affecting healthy cells [3]. |
| 4 | N-Nonanoylglycine | Metabolomic studies have identified this compound as a biomarker for various cancers [4]. |
| 5 | 2-Hydroxyestrone | Circulating levels of this metabolite have been shown to be associated with breast and endometrial cancer, although its role in cervical cancer remains to be investigated [5]. |
| 6 | 3b-Hydroxy-5-cholenoic acid | Has been detected as a component of the human urine metabolome, and is associated with cancer risk [6]. |
| 7 | Phosphoenolpyruvic acid | This metabolite has been identified as a metabolic checkpoint of anti-tumor T cell responses [7]. |
| 8 | Cholesterol sulfate | This metabolite is highly expressed in tissues with well-differentiated cervical carcinoma, and other squamous cell cancers [8]. |
| 9 | Cortisol | Cortisol is a biomarker for stress, and its levels are elevated in cervical cancer patients [9]. |
| 10 | Cholesterol sulfate | This metabolite is highly expressed in tissues with well-differentiated cervical carcinoma, and other squamous cell cancers [10]. |
Table S6: Select metabolites involved in the signature for distinguishing cervical cancer from the other women-specific cancers

## Slide 9
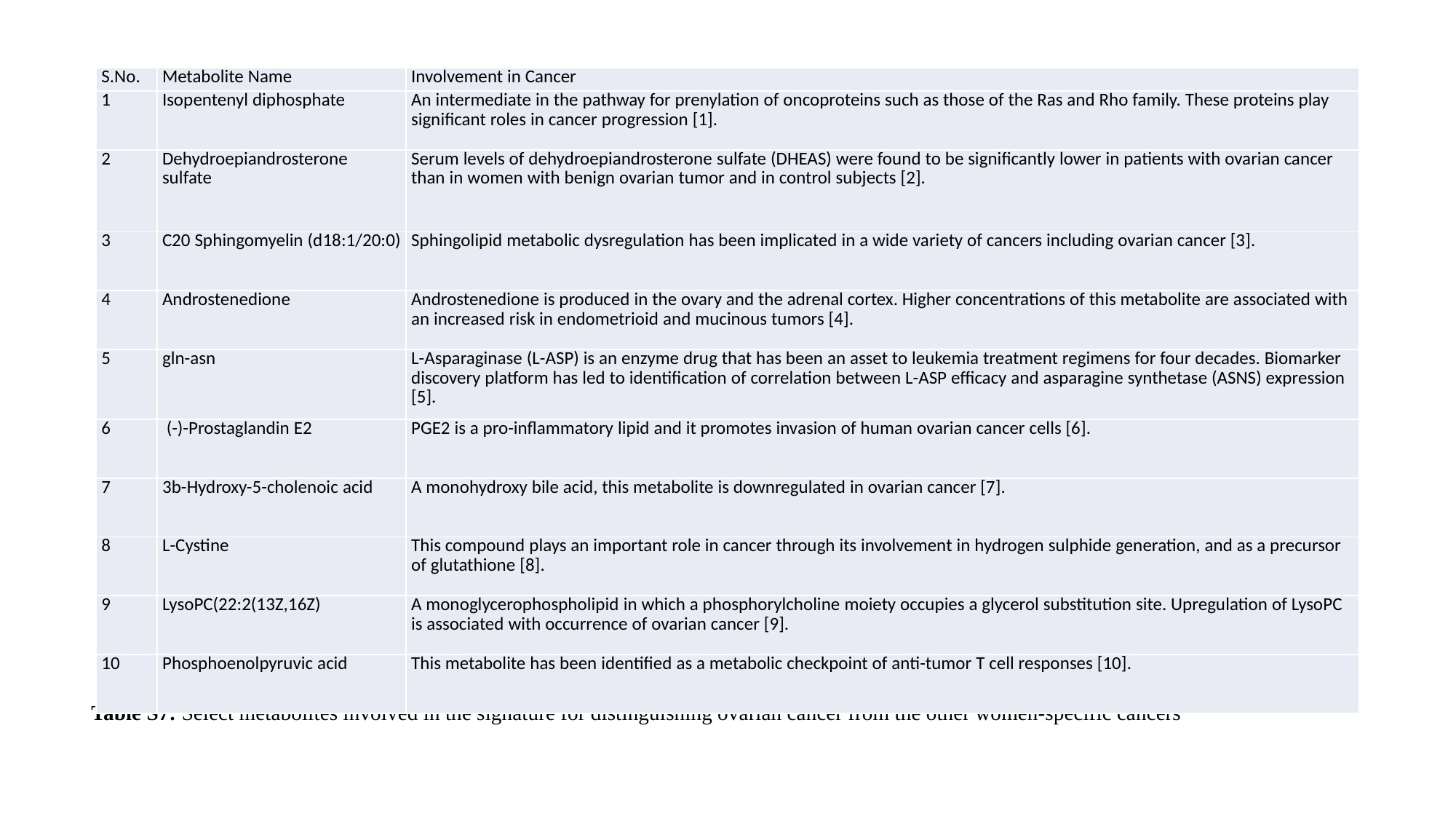

| S.No. | Metabolite Name | Involvement in Cancer |
| --- | --- | --- |
| 1 | Isopentenyl diphosphate | An intermediate in the pathway for prenylation of oncoproteins such as those of the Ras and Rho family. These proteins play significant roles in cancer progression [1]. |
| 2 | Dehydroepiandrosterone sulfate | Serum levels of dehydroepiandrosterone sulfate (DHEAS) were found to be significantly lower in patients with ovarian cancer than in women with benign ovarian tumor and in control subjects [2]. |
| 3 | C20 Sphingomyelin (d18:1/20:0) | Sphingolipid metabolic dysregulation has been implicated in a wide variety of cancers including ovarian cancer [3]. |
| 4 | Androstenedione | Androstenedione is produced in the ovary and the adrenal cortex. Higher concentrations of this metabolite are associated with an increased risk in endometrioid and mucinous tumors [4]. |
| 5 | gln-asn | L-Asparaginase (L-ASP) is an enzyme drug that has been an asset to leukemia treatment regimens for four decades. Biomarker discovery platform has led to identification of correlation between L-ASP efficacy and asparagine synthetase (ASNS) expression [5]. |
| 6 | (-)-Prostaglandin E2 | PGE2 is a pro-inflammatory lipid and it promotes invasion of human ovarian cancer cells [6]. |
| 7 | 3b-Hydroxy-5-cholenoic acid | A monohydroxy bile acid, this metabolite is downregulated in ovarian cancer [7]. |
| 8 | L-Cystine | This compound plays an important role in cancer through its involvement in hydrogen sulphide generation, and as a precursor of glutathione [8]. |
| 9 | LysoPC(22:2(13Z,16Z) | A monoglycerophospholipid in which a phosphorylcholine moiety occupies a glycerol substitution site. Upregulation of LysoPC is associated with occurrence of ovarian cancer [9]. |
| 10 | Phosphoenolpyruvic acid | This metabolite has been identified as a metabolic checkpoint of anti-tumor T cell responses [10]. |
Table S7: Select metabolites involved in the signature for distinguishing ovarian cancer from the other women-specific cancers
