# Supplementary material for: A non-invasive method for concurrent detection of early-stage women-specific cancers
Source: Sci Rep. 2022 Feb 10;12:2301. doi: 10.1038/s41598-022-06274-9 (PMC8831619; doi:10.1038/s41598-022-06274-9)
Supplement: Supplementary file 3 — Supplementary Information. [file 41598_2022_6274_MOESM3_ESM.docx]

**A NON-INVASIVE METHOD FOR CONCURRENT DETECTION OF EARLY-STAGE WOMEN-SPECIFIC CANCERS**

**Ankur Gupta^1#^, Ganga Sagar^1#^, Zaved Siddiqui^1^, Kanury V.S. Rao^1,2^, Sujata Nayak^1,2^, Najmuddin Saquib^1*^ & Rajat Anand^1,2,*^**

**^1^PredOmix Technologies Private Limited**

**Tower B, SAS Tower**

**Medicity, Sector – 38**

**Gurugram – 122002,**

**India**

**^2^PredOmix, Inc.**

**9853 Pacific Heights Blvd.**

**San Diego, CA 92121-4721**

**USA**

^#^These authors contributed equally to this work.

^*^Address correspondence and requests for materials to RA (email: [rajat@predomix.com](mailto:rajat@predomix.com)) or NS (email: saquib@predomix.com)

|  | **Top 100 ranked metabolites for each classification** | | |
| --- | --- | --- | --- |
|  | 1. **BECO vs Normal** | 1. **Breast vs ECO** | 1. **Endometrial vs BCO** |
| 1 | Nisinic acid | 2-Hydroxyestrone | N-LINOLEOYL-4-AMINOBUTYRIC ACID |
| 2 | Formiminoglutamic Acid | isopentenyl diphosphate | 1,3-Bis[(9Z)-9-tetradecenoyloxy]-2-propanyl (6Z,9Z,12Z,15Z)-6,9,12,15-octadecatetraenoate |
| 3 | 2-[(5Z,8Z,11Z,14Z,17Z)-eicosapentaenoyl]-sn-glycerol | Asp-Gln | 3-(3,4-Dimethoxyphenyl)-N-[2-(3,4-dimethoxyphenyl)-2-hydroxyethyl]propanamide |
| 4 | (3,3b,5',6-Tetramethyl-8-oxoicosahydrospiro[furo[2'',3'':4',5']cyclopenta[1',2':7,8]naphtho[2,3-b]furan-2,2'-pyran]-6-yl)acetic acid | Biliverdin | 2-Amino-2-deoxy-alpha-D-glucopyranosyl-(1->4)-2-O-sulfo-alpha-L-idopyranuronosyl-(1->4)-2-deoxy-6-O-sulfo-2-(sulfoamino)-alpha-D-glucopyranose |
| 5 | Androsterone glucuronide | 2-(Asparaginylamino)-4-carboxybutanoate | arg-his |
| 6 | 9a-Methoxy-3,4a,5-trimethyl-6-{[(2E)-3-(methylsulfanyl)-2-propenoyl]oxy}-2-oxo-2,4,4a,5,6,7,8,8a,9,9a-decahydronaphtho[2,3-b]furan-4-yl (2E)-2-methyl-2-butenoate | Arg-Pro | PG(18:1(11Z)/18:2(9Z,12Z)) |
| 7 | 3,4,15-Triacetoxy-12,13-epoxytrichothec-9-en-8-yl 3-methylbutanoate | 4-Chlorodehydromethyltestosterone | D-Tyrosyl-L-alloisoleucyl-D-leucine |
| 8 | 1-O-[(3beta,5xi,9xi,19alpha)-3-(alpha-L-Arabinopyranosyloxy)-19-hydroxy-28-oxoolean-12-en-28-yl]-beta-D-glucopyranose | LysoPC(22:2(13Z,16Z)) | 1-arachidonoyl-sn-glycerol 3-phosphate |
| 9 | 3-sec-Butyl-9-(1-hydroxyethyl)-6,15-diisopropyl-12-methyl-1,7-dioxa-4,10,13-triazacyclopentadecane-2,5,8,11,14-pentone | 1-oleoyl-2-arachidonoyl-sn-glycero-3-phospho-L-serine | N-Methyl-5-[(1-pyrrolidinylsulfonyl)methyl]-1H-indole-3-ethanamine |
| 10 | 3448 | 5-Oxoprolylprolyl-N-[14-carboxy-11-(hydroxymethyl)-5-isopropyl-3,6,9,12-tetraoxo-1,4,7,10,13-pentaazatricyclo[14.6.1.0~17,22~]tricosa-16(23),17,19,21-tetraen-2-yl]tryptophanamide | (3R,3aR,5S,5aR,5bR,9S,11aR,13aR,13bS)-5-Hydroxy-3a,5a,5b,8,8,11a-hexamethyl-3-(3-methyl-2-buten-1-yl)-1-oxooctadecahydro-1H-furo[3,4-c]naphtho[1,2-h]isochromen-9-yl 6-O-(6-deoxy-alpha-L-mannopyranosyl )-beta-D-glucopyranoside |
| 11 | 3-Methoxytyramine | (1Z)-1-(4-Hydroxy-3-methoxyphenyl)-1-decene-3,5-dione | LTF4 |
| 12 | LysoPC(P-18:0) | 2-Butenedioic acid (2E)-, (2E,6E,10E,14E)-2,6,10,14-tetramethyl-2,6,10,14-hexadecatetraene-1,16-diyl ester | 9-[5-O-(Hydroxy{[hydroxy(phosphonooxy)phosphoryl]oxy}phosphoryl)pentofuranosyl]-6-methylene-6,9-dihydro-1H-purin-2-amine |
| 13 | Varanic acid | 8,8-Dimethyl-3-(2-methyl-3-buten-2-yl)-10-(3-methyl-2-buten-1-yl)-2H,8H-pyrano[3,2-g]chromen-2-one | 1-stearyl estercitric acid |
| 14 | 8-Amino-7-oxononanoic acid | 1-(16,17-Dimethoxyaspidospermidin-1-yl)ethanone | Cys-Asn |
| 15 | L-(+)-glutamine | (2S)-2-(Palmitoyloxy)-3-(pentadecanoyloxy)propyl (4Z,7Z,10Z,13Z,16Z)-4,7,10,13,16-docosapentaenoate | O-[(2-{[13-(3,4-Dimethyl-5-pentyl-2-furyl)tridecanoyl]oxy}-3-{[11-(3,4-dimethyl-5-pentyl-2-furyl)undecanoyl]oxy}propoxy)(hydroxy)phosphoryl]serine |
| 16 | Methyl (3S)-3-[(3S,3aS,6R,7S,9R,9aS,9bR,10aS)-3-(3-furyl)-9-hydroxy-7-(2-hydroxy-2-propanyl)-3a,6,9a-trimethyl-1,8-dioxododecahydrobenzo[f]oxireno[d]isochromen-6-yl]-3-hydroxypropanoate | 4-[(1Z)-4-Chloro-1,2-diphenyl-1-buten-1-yl]phenol | 2H-Pyrazino(1',2':1,5)pyrrolo(2,3-b)indole-1,4(3H,5aH)-dione, 10b-(1,1-dimethyl-2-propenyl)-6,10b,11,11a-tetrahydro-3-(1H-imidazol-4-ylmethylene)- |
| 17 | Serotonin | epsilon-(gamma-Glutamyl)-lysine | D-Arginyl-L-arginyl-D-isoleucine |
| 18 | 4-O-{3-O-[(1S,2R,3S,4S,5R)-2,3,4-Trihydroxy-5-(hydroxymethyl)cyclohexyl]-beta-D-galactopyranosyl}-D-glucose | S(8)-(2-methylbutanoyl)dihydrolipoamide | 1-octadecanoyl-2-[(4Z,7Z,10Z,13Z,16Z)-docosapentaenoyl]-sn-glycero-3-phosphoethanolamine |
| 19 | LysoPC(22:2(13Z,16Z)) | KG7537500 | 2'-Hydroxy-5'',6''-dimethoxy-13,14-dihydrolythran-12-one |
| 20 | 6-Acetamido-2-oxohexanoic acid | 18-Hydroxycortisol | O-{[(2R)-2-[(13Z)-13-Docosenoyloxy]-3-(pentadecanoyloxy)propoxy](hydroxy)phosphoryl}-L-serine |
| 21 | Adenylthiomethylpentose | 7Î±-Hydroxy-3-oxo-4-cholen-24-oic acid | 18-Oxocortisol |
| 22 | Pyridoxamine 5'-phospate | 1-myristoyl-2-palmitoleoyl-sn-glycero-3-phosphocholine | O-heptadecanoylcarnitine |
| 23 | MFCD00065147 | 5,7-Dihydro-4H-purin-8-ol | FAD |
| 24 | 4-{(Z)-[(4E,7Z,16Z,19Z)-1-Hydroxy-4,7,10,13,16,19-docosahexaen-1-ylidene]amino}butanoic acid | N-Acetylserotonin sulfate | N-Nonanoylglycine |
| 25 | 1-(4-Hydroxy-3-methoxyphenyl)-3-oxo-5-decanesulfonic acid | gamma-Glutamyl-3-(2-methylenecyclopropyl)alanine | 24-Hydroxycalcitriol |
| 26 | Methyl 5,7,11-trihydroxy-2-methyl-2',10-dioxospiro[9-oxatricyclo[6.3.1.0~1,5~]dodecane-6,3'-oxetane]-7-carboxylate | 3',5',3-Triiodothyronine | pro-met |
| 27 | 2-Hydroxy-4-(4-methoxyphenyl)-1H-phenalen-1-one | Lys-Tyr | (2R)-1-(Phosphonooxy)-3-(stearoyloxy)-2-propanyl (4Z,7Z,10Z,13Z,16Z)-4,7,10,13,16-docosapentaenoate |
| 28 | gamma-Glutamyl-S-propylcysteine | (2S)-3-(4-Hydroxyphenyl)-2-({[(3S,4S,5R)-2,3,4-trihydroxy-5-(hydroxymethyl)tetrahydro-2-furanyl]methyl}amino)propanoic acid (non-preferred name) | 2-({(2E)-3-[(2Z)-2-(3,4-Dihydroxybenzylidene)-3-oxo-2,3-dihydro-1,4-benzodioxin-6-yl]-2-propenoyl}oxy)-3-(3,4-dihydroxyphenyl)propanoic acid |
| 29 | 4a-Hydroxy-12-methoxy-4,4,6a,8,13b-pentamethyl-1,4,4a,5,6,6a,9,13,13a,13b-decahydro-2H-benzo[a]furo[3,4-i]xanthene-3,11-dione | (5Z)-5-{[(1Z,2R)-1-[(Carboxymethyl)imino]-1-hydroxy-3-{[(8alpha,9beta,14beta,17beta)-3,4,17-trihydroxyestra-1(10),2,4-trien-2-yl]sulfanyl}-2-propanyl]imino}-5-hydroxy-L-norvaline | D-Isoleucyl-L-prolyl-L-tyrosyl-D-isoleucine |
| 30 | L-Proline | Hippuric acid | O-{[(2R)-2-[(4Z,7Z,10Z,13Z,16Z,19Z)-4,7,10,13,16,19-Docosahexaenoyloxy]-3-(tetradecanoyloxy)propoxy](hydroxy)phosphoryl}-L-serine |
| 31 | DIACETIN MONOPROPANOATE | UROBILIN, (-)- | UROBILIN, (-)- |
| 32 | N-Acetyl-S-{[4-(methylsulfinyl)butyl]carbamothioyl}cysteine | 24-Hydroxycalcitriol | n-Butyl lactate |
| 33 | MHPG | Androsterone glucuronide | (6-{6-[5'-Ethyl-2'-hydroxy-5'-(1-hydroxyethyl)-2,3',4-trimethyloctahydro-2,2'-bifuran-5-yl]-3-hydroxy-4-methyl-5-oxo-2-octanyl}-2-hydroxy-3,5-dimethyltetrahydro-2H-pyran-2-yl)acetic acid |
| 34 | 3-(2,6-Dioxo-3,6-dihydro-1(2H)-pyrimidinyl)alanine | 2,6,8-Trihydroxy-4-isopropyl-3,7,11-trimethyl-14-oxo-13-oxatetracyclo[5.5.3.0~1,8~.0~2,6~]pentadec-3-en-12-yl acetate | (8E)-2-Amino-8-octadecene-1,3,4-triol |
| 35 | Platelet-activating factor | Thromboxane B2 | Ethyl (2R)-2-[(3S,5aS,9aR,10aS)-3-methyl-1,4-dioxodecahydropyrazino[1,2-a]indol-2(1H)-yl]-4-phenylbutanoate |
| 36 | 5-Methylcytidine | 1-palmitoylglycerone 3-phosphate | Serotonin |
| 37 | 2-Amino-6-[(E)-(5-amino-5-carboxy-2-hydroxypentylidene)amino]-5-hydroxyhexanoic acid (non-preferred name) | Testosterone glucuronide | MIPC |
| 38 | 2-Isopropyl-5-methylcyclohexyl beta-D-glucopyranosiduronic acid | beta-Aspartylaspartic acid | His-His |
| 39 | 1-Methylinosine | Dolichyl b-D-glucosyl phosphate | 3-hydroxytetradecanoylcarnitine |
| 40 | Lewis x | PC(18:4(6Z,9Z,12Z,15Z)/P-18:1(11Z)) | D-Arginyl-L-histidyl-D-prolyl-D-tyrosine |
| 41 | L-Prolyl-L-prolyl-L-lysyl-N~5~-(diaminomethylene)-L-ornithine | 1-arachidoyl-2-arachidonoyl-sn-glycero-3-phosphate | N-Acetylglutamine |
| 42 | Pro-tyr | Testosterone | 1-Isothiocyanato-8-(methylthio)octane |
| 43 | protoporphyrinogen | Ceramide (d18:1/9Z-18:1) | (2E,2'E)-3,3'-[2,4-Bis(1,3-benzodioxol-5-yl)-1,3-cyclobutanediyl]bis[1-(1-piperidinyl)-2-propen-1-one] |
| 44 | 9-[5-O-(Hydroxy{[hydroxy(phosphonooxy)phosphoryl]oxy}phosphoryl)pentofuranosyl]-6-methylene-6,9-dihydro-1H-purin-2-amine | 1-Myristoyl-2-hydroxy-sn-glycero-3-PE | N-Heptadecanoylglycine |
| 45 | 2-Cyano-3-(3,4-dihydroxy-5-nitrophenyl)-N,N-diethylpropanamide | 3-(2-Acetamidoethyl)-1-hexopyranuronosyl-5-methoxy-1H-indole | 20-hydroxy-leukotriene E4 |
| 46 | 4-Amino-1-[(2xi)-5-O-{hydroxy[(hydroxy{(2R)-2-[(12-methyltridecanoyl)oxy]-3-[(9Z,11Z)-9,11-octadecadienoyloxy]propoxy}phosphoryl)oxy]phosphoryl}-beta-D-threo-pentofuranosyl]-2(1H)-pyrimidinone | 1Î²-Hydroxycholic acid | (2S,8R)-2-Ammonio-5-hydroxy-10-oxo-8-[(pentadecanoyloxy)methyl]-4,6,9-trioxa-5-phosphaheptacosan-1-oate 5-oxide |
| 47 | 13,14-Dihydroretinol | 7-Hydroxy-9-methoxy-6-(1,3,4-trihydroxy-2-butanyl)-1,2-dihydrocyclopenta[c]chromene-3,4-dione | (2R)-1-(Icosanoyloxy)-3-(phosphonooxy)-2-propanyl (4Z,7Z,10Z,13Z,16Z,19Z)-4,7,10,13,16,19-docosahexaenoate |
| 48 | 19-Methoxy-8-[(4E)-6-methoxy-6-methyl-4-hepten-2-yl]-5,9,17,17-tetramethyl-18-oxapentacyclo[10.5.2.0~1,13~.0~4,12~.0~5,9~]nonadec-2-en-16-yl hexopyranoside | (2S)-3-Hydroxy-1,2-propanediyl (7Z,10Z,13Z,16Z,19Z,7'Z,10'Z,13'Z,16'Z,19'Z)bis(-7,10,13,16,19-docosapentaenoate) | (2R)-3-{[(2-Aminoethoxy)(hydroxy)phosphoryl]oxy}-2-[(1Z)-1-octadecen-1-yloxy]propyl icosanoate |
| 49 | 4-[(2R)-2-(Aminomethyl)-2-(hydroxymethyl)-5-oxo-1-pyrrolidinyl]-3-(3-pentanylamino)benzoic acid | (2R)-3-[(Hydroxy{[(1s,3R)-2,3,4,5,6-pentahydroxycyclohexyl]oxy}phosphoryl)oxy]-1,2-propanediyl dihexadecanoate | Lacto-N-fucopentaose-2 |
| 50 | 1-(4-Hydroxy-3-methoxyphenyl)-3,5-decanediyl diacetate | (8beta,11alpha,15R)-16,16-Difluoro-15-hydroxy-9-oxo-11,15-epoxyprostan-1-oic acid | 2-Phenylaminoadenosine |
| 51 | 1,2-Diarachidonoyl-sn-glycero-3-PC | spermidine | 1-heptadecanoyl-sn-glycero-3-phosphate |
| 52 | Methyl 6,10b-dihydroxy-2,4b,7,7,10a,12a-hexamethyl-12-methylene-1,4,5,8-tetraoxo-1,4,4a,4b,5,7,8,9,10,10a,10b,11,12,12a-tetradecahydro-2H-naphtho[1,2-h]isochromene-2-carboxylate | 3-{(2Z)-2-{2-[(3-Ethyl-5-formyl-4-methyl-1H-pyrrol-2-yl)methyl]-5-(methoxycarbonyl)-3-methyl-4-oxo-4,5-dihydrocyclopenta[b]pyrrol-6(1H)-ylidene}-4-methyl-5-[(3-methyl-5-oxo-4-vinyl-2,5-dihydro-1H-pyrr ol-2-yl)methyl]-3,4-dihydro-2H-pyrrol-3-yl}propanoic acid | (5alpha,6beta)-3-Hydroxy-17-methyl-4,5-epoxymorphinan-6-yl beta-L-glucopyranosiduronic acid |
| 53 | 2-Oxo-1,2-diphenylethyl hydrogen sulfate | Endomorphin 2 | Vitamin C |
| 54 | 12,13-Epoxytrichothec-9-ene-3,4,8,15-tetrol | 1-stearoyl-2-(4Z,7Z,10Z,13Z,16Z,19Z)-docosahexaenoyl-sn-glycero-3-phosphate | 7-[(3,3-Dimethyl-2-oxiranyl)methyl]-4-hydroxy-2-(2-hydroxy-2-propanyl)-5-isobutyryl-3a-(3-methyl-2-buten-1-yl)-3,3a-dihydro-1-benzofuran-6(2H)-one |
| 55 | 17-beta-Estradiol | N-Methyl-5-[(1-pyrrolidinylsulfonyl)methyl]-1H-indole-3-ethanamine | 1-(Octadecyloxy)-3-(pentadecanoyloxy)-2-propanyl stearate |
| 56 | Methyl [5,11-diacetoxy-13-(3-furyl)-16-hydroxy-6,6,8,12-tetramethyl-17-methylene-15-oxo-2,14-dioxatetracyclo[7.7.1.0~1,12~.0~3,8~]heptadec-7-yl](hydroxy)acetate | 5-Formiminotetrahydrofolic acid | 7-(2,3-Dihydroxy-3-methylbutoxy)-8-(3-methyl-2-oxobutyl)-2H-chromen-2-one |
| 57 | 10a-Hydroxy-5-methoxy-3,4,7a,10a-tetrahydro-1H,12H-furo[3',2':4,5]furo[2,3-h]pyrano[3,4-c]chromene-1,12-dione | 4-(1-Hydroxy-2-{[6-(4-hydroxy-4-phenylbutoxy)hexyl]amino}ethyl)-2-(hydroxymethyl)phenol | 1-(1Z-hexadecenyl)-2-arachidonoyl-sn-glycero-3-phosphoethanolamine |
| 58 | 7-ketodeoxycholic acid | 3-Thiohexyl acetate | 3',5',3-Triiodothyronine |
| 59 | 2-Aminoethyl (2R)-3-[(1Z)-1-hexadecen-1-yloxy]-2-hydroxypropyl hydrogen phosphate | Methionylleucine | Linoleamide |
| 60 | N-(2-Phenylethyl)-beta-D-glucopyranuronosylamine | PG(16:0/22:6(4Z,7Z,10Z,13Z,16Z,19Z)) | N-lauroylglycine |
| 61 | PAF C-18:1 | 1,1â€²-[1-[(Phosphonooxy)methyl]-1,2-ethanediyl] di-(9Z,12Z,15Z)-9,12,15-octadecatrienoate | O-(Hydroxy{(2R)-3-[(5Z,8Z,11Z)-5,8,11-icosatrienoyloxy]-2-[(15Z)-15-tetracosenoyloxy]propoxy}phosphoryl)-L-serine |
| 62 | Androstenedione | 5-[3-(Methylamino)propyl]-10,11-dihydro-5H-dibenzo[b,f]azepin-2-yl beta-D-glucopyranosiduronic acid | Ceramide (d18:1/20:0) |
| 63 | (3R,4S,5S,6R,7R,9R,10E,11S,12R,13S,14R)-14-Ethyl-7,12,13-trihydroxy-4-{[(2R,5S)-5-hydroxy-4-methoxy-6-methyltetrahydro-2H-pyran-2-yl]oxy}-6-{[(2S,6R)-3-hydroxy-6-methyl-4-(methylamino)tetrahydro-2H-py ran-2-yl]oxy}-10-{[(2-methoxyethoxy)methoxy]imino}-3,5,7,9,11,13-hexamethyloxacyclotetradecan-2-one (non-preferred name) | 1-octadecanoyl-2-(7Z,10Z,13Z,16Z-docosatetraenoyl)-sn-glycero-3-phosphocholine | 1-(16,17-Dimethoxyaspidospermidin-1-yl)ethanone |
| 64 | 6-Sulfatoxymelatonin | 1-palmitoyl-2-stearoyl-sn-glycero-3-phosphoserine | 4-O-{3-O-[(1S,2R,3S,4S,5R)-2,3,4-Trihydroxy-5-(hydroxymethyl)cyclohexyl]-beta-D-galactopyranosyl}-D-glucose |
| 65 | 3-[2-(3,4-Dihydroxy-5-methoxyphenyl)ethyl]phenyl hydrogen sulfate | 3-Deoxy-D-glycero-D-galacto-2-nonulosonic Acid | DV5460000 |
| 66 | 1-(1Z-hexadecenyl)-sn-glycero-3-phosphocholine | (2E,2'E)-3,3'-[2,4-Bis(1,3-benzodioxol-5-yl)-1,3-cyclobutanediyl]bis[1-(1-piperidinyl)-2-propen-1-one] | Cholesteryl palmitoleate |
| 67 | 5,6-Dihydroxy-7-methoxy-2,2-dimethyl-3,4-dihydro-2H-chromen-4-yl hydrogen sulfate | N-Nonanoylglycine | 10-[4-(2,4,4-Trimethyl-2-pentanyl)phenoxy]-1-decanol |
| 68 | GUDCA | 5,7-Dimethoxy-2',2'-dimethyl-6-(3-methyl-2-buten-1-yl)-3,4-dihydro-2H,2'H-3,8'-bichromen-5'-ol | PC(18:3(6Z,9Z,12Z)/P-18:1(11Z)) |
| 69 | arg-cys | (2-Hydroxy-2-oxido-1,3,2-dioxaphospholan-4-yl)methyl (9Z)-9-octadecenoate | Palmitoleoyl ethanolamide |
| 70 | (2R)-2-Hydroxy-3-(phosphonooxy)propyl (5Z,8Z,11Z,14E,17E)-5,8,11,14,17-icosapentaenoate | Phenyl D-glucopyranosiduronic acid | Gln-Asn |
| 71 | 4-Hydroxy-5-methyl-3(2H)-thiophenone | Lys-Thr | beta-D-Glucopyranuronosyl-(1->3)-(3xi)-2-acetamido-2-deoxy-beta-D-ribo-hexopyranosyl-(1->4)-beta-D-glucopyranuronosyl-(1->3)-(3xi)-2-acetamido-2-deoxy-beta-D-ribo-hexopyranose |
| 72 | N-Acetyl-DL-Histidine | 5,8-Dihydroxy-2-(1-hydroxy-3-methoxy-4-oxocyclohexyl)-3,7-dimethoxy-4H-chromen-4-one | N-{[3-Ethyl-11,15-dihydroxy-7-isopropyl-3-methyl-10-(methylamino)-6,9-dioxo-2-oxa-5,8-diazabicyclo[10.3.1]hexadeca-1(16),12,14-trien-4-yl]carbonyl}glycine |
| 73 | (1aR,2R,3S,11cS)-6,11-Dimethyl-1a,2,3,11c-tetrahydrotetrapheno[1,2-b]oxirene-2,3-diol | palmitoleoyl ethanolamide | 8-[(4E)-6-Hydroxy-6-methyl-4-hepten-2-yl]-19-methoxy-5,9,17,17-tetramethyl-18-oxapentacyclo[10.5.2.0~1,13~.0~4,12~.0~5,9~]nonadec-2-en-16-yl hexopyranoside |
| 74 | 3,4-Dimethoxyphenethylamine | N6-threonylcarbamoyladenosine | urobilinogen |
| 75 | 12(13)Ep-9-KODE | (7R,9Z,11R,12S,13R,14S,15S,16S,17R,18R,19Z,21Z)-2,13,15,17,27,29-Hexahydroxy-11-methoxy-3,7,12,14,16,18,22-heptamethyl-26-{(Z)-[(4-methyl-1-piperazinyl)imino]methyl}-8,30-dioxa-24-azatetracyclo[23.3.1 .1~4,7~.0~5,28~]triaconta-1(29),2,4,9,19,21,25,27-octaene-6,23-dione | L-Tyrosyl-L-prolyl-D-glutaminyl-L-prolyl-L-glutaminyl-D-prolyl-D-phenylalanine |
| 76 | O-heptadecanoylcarnitine | O-{[(2R)-2-[(7Z,10Z,13Z,16Z)-7,10,13,16-Docosatetraenoyloxy]-3-(pentadecanoyloxy)propoxy](hydroxy)phosphoryl}-L-serine | Guanidinosuccinic acid |
| 77 | Leukotriene D4 | (4E)-7-(4-Hydroxy-3,5-dimethoxyphenyl)-1-(4-hydroxy-3-methoxyphenyl)-4-hepten-3-one | 7-Methyl-2,6,9,12-tetraoxahexadecane |
| 78 | 2-Hydroxy-N-{2-[2-(1H-imidazol-5-yl)ethyl]-5-methyl-3-oxo-1,2-oxazolidin-4-yl}benzamide | 15,16-DiHODE | 1-(4-Hydroxy-3,5-dimethoxyphenyl)-7-(4-hydroxy-3-methoxyphenyl)-3,5-heptanediol |
| 79 | 1,4'-Bipiperidine-1'-carboxylic acid | 2-(1-Hydroxy-2,4,6-trimethyl-3-oxo-2,3-dihydro-1H-inden-5-yl)ethyl acetate | 4-(2,2'-Bithiophen-5-yl)-3-butyn-1-ol |
| 80 | LTF4 | (5Z,7E,14xi,22E)-3-Hydroxy-9,10-secoergosta-5,7,10,22-tetraen-25-yl beta-D-glucopyranosiduronic acid | Prostaglandin D2 Ethanolamide |
| 81 | lysophosphatidylethanolamine (22:6(4Z,7Z,10Z,13Z,16Z,19Z)/0:0) | (4R,8S)-11-[(2R)-6-Hydroxy-2,5,7,8-tetramethyl-3,4-dihydro-2H-chromen-2-yl]-4,8-dimethylundecanoic acid | 3-Hydroxyhexadecadienoylcarnitine |
| 82 | Glycylprolylhydroxyproline | 4-(4-Chlorophenyl)-1-[4-(4-fluorophenyl)-4-oxobutyl]-4-piperidinyl D-glucopyranosiduronic acid | 5,6,14-Trihydroxy-1,26-dioxo-16,17:22,26-diepoxyergosta-2,24-dien-15-yl acetate |
| 83 | spiro[8-azoniabicyclo[3.2.1]octane-8,1'-azolidin-1-ium]-3-yl 2-hydroxy-2,2-diphenyl-acetate | pro-met | DL-Lactic Acid |
| 84 | (5Z)-14-{[(2E)-3-(4-Hydroxyphenyl)-2-propenoyl]oxy}-5-tetradecene-8,10,12-triynoic acid | Alpha-Aminoadipic acid | 4-Methylene-2-oxoglutarate |
| 85 | 4-(4-Chlorophenyl)-1-[4-(4-fluorophenyl)-4-oxobutyl]-4-piperidinyl D-glucopyranosiduronic acid | Dexamethasone | Glycylglutamine |
| 86 | (3alpha,5beta,7alpha,12beta)-7,12-Dihydroxy-24-oxo-24-[(2-sulfoethyl)amino]cholane-3-sulfonic acid | Deoxycholic acid | 2,4-Dimethyl-3-pentanamine |
| 87 | asp-gln | 4-[(2-Hydroxy-4-methylpentanoyl)amino]-7-isobutyl-3-isopropyl-5,8-dioxo-2-oxa-6,9-diazabicyclo[10.2.2]hexadeca-1(14),12,15-triene-10-carboxylic acid | 6,9,11,13,14-Pentahydroxy-11-isopropyl-3,7,10-trimethyl-15-oxapentacyclo[7.5.1.0~1,6~.0~7,13~.0~10,14~]pentadec-2-yl acetate |
| 88 | lys-tyr | 1-octadecanoyl-2-(7Z,10Z,13Z,16Z-docosatetraenoyl)-sn-glycero-3-phosphocholine | L-Homocysteic acid |
| 89 | N-Gluconyl ethanolamine phosphate | 2-Hydroxy-3-(phosphonooxy)propyl (4Z,7Z,10Z,13Z,16Z)-4,7,10,13,16-docosapentaenoate | 2-Hydroxyestrone |
| 90 | Dihydropteroic acid | 6,9a-Dihydroxy-3,4a,5-trimethyl-2-oxo-2,4,4a,5,6,7,8,8a,9,9a-decahydronaphtho[2,3-b]furan-4-yl 3-chloro-2-hydroxy-2-methylbutanoate | 3-Isopropyl-7-methyl-8-(4-methyl-4-penten-1-yl)-1,4-dioxo-1,4-dihydro-2-naphthalenyl acetate |
| 91 | (8E,22E)-4-Methoxy-2-oxa-11,16,20-triazatricyclo[22.2.2.1~3,7~]nonacosa-1(26),3(29),4,6,8,22,24,27-octaene-10,21-dione | 2-Hydroxyestrone | (8beta,11alpha,15R)-16,16-Difluoro-15-hydroxy-9-oxo-11,15-epoxyprostan-1-oic acid |
| 92 | (2R)-1-(Phosphonooxy)-3-[(9Z)-9-tetradecenoyloxy]-2-propanyl (6Z,9Z,12Z,15Z)-6,9,12,15-octadecatetraenoate | (1aR,2R,3S,11cS)-6,11-Dimethyl-1a,2,3,11c-tetrahydrotetrapheno[1,2-b]oxirene-2,3-diol | 1,2-dioleoyl-sn-glycero-3-phospho-N,N-dimethylethanolamine |
| 93 | 5-Hydroxy-1,7-diphenyl-6-hepten-3-one | (3alpha,5beta,7alpha,12beta)-7,12-Dihydroxy-24-oxo-24-[(2-sulfoethyl)amino]cholane-3-sulfonic acid | (2R)-2-[(5Z,8Z,11Z,14Z,17Z)-5,8,11,14,17-Icosapentaenoyloxy]-3-[(6Z,9Z,12Z)-6,9,12-octadecatrienoyloxy]propyl 2-(trimethylammonio)ethyl phosphate |
| 94 | 2,3-Bis(4-hydroxy-3-methoxybenzyl)-1,2,4-butanetriol | 6,17-Dihydroxy-16-methyl-8-(2-methyl-2-propanyl)-2,4,14,19-tetraoxahexacyclo[8.7.2.0~1,11~.0~3,7~.0~7,11~.0~13,17~]nonadecane-5,15,18-trione | Tyramine |
| 95 | 3-[2-(3,5-Dihydroxyphenyl)ethyl]phenyl hydrogen sulfate | asp-gln | 1-Palmitoyl-2-linoleoyl PE |
| 96 | 6-Methoxy-3-(2-thiazolyl)-1H-indole | (4Z,7Z,10Z,19Z)-14-[(4Z,7Z,10Z,13Z,16Z,19Z)-4,7,10,13,16,19-Docosahexaenoyloxy]-4,7,10,12,16,19-docosahexaenoic acid | 1-linoleyl-sn-glycerol 3-phosphate |
| 97 | 4-(Methylthio)-2-oxobutyric acid | (2R)-1-(Pentadecanoyloxy)-3-(phosphonooxy)-2-propanyl (13Z)-13-docosenoate | (15R,21S)-18,21,24,24-Tetrahydroxy-3-methyl-18,24-dioxido-12-oxo-13,17,19,23-tetraoxa-18lambda~5~,24lambda~5~-diphosphatetracosan-15-yl (9Z,11Z)-9,11-octadecadienoate |
| 98 | 1-(4-Hydroxybenzyl)-7,8-dimethoxy-2,2-dimethyl-1,2,3,4-tetrahydroisoquinolinium | (14R,20S)-17,20,23,23-Tetrahydroxy-2-methyl-17,23-dioxido-11-oxo-12,16,18,22-tetraoxa-17lambda~5~,23lambda~5~-diphosphatricosan-14-yl (9Z,11Z)-9,11-octadecadienoate | 1-hexadecanoyl-2-(5Z,8Z,11Z,14Z-icosatetraenoyl)-sn-glycero-3-phosphoethanolamine |
| 99 | 2-Isopropyl-3,5-dimethoxy-6-methylpyrazine | 4-(Hydroxy{7-hydroxy-6-methoxy-8-[(6-methoxy-2-methyl-1,2,3,4-tetrahydro-7-isoquinolinyl)oxy]-2-methyl-1,2,3,4-tetrahydro-1-isoquinolinyl}methyl)-1,2-benzenediol | 1-linoleyl-sn-glycerol 3-phosphate |
| 100 | 1-(4-Hydroxy-3,5-dimethoxyphenyl)-7-(4-hydroxy-3-methoxyphenyl)-3,5-heptanediol | 4-Oxo-4-(1H-purin-6-ylamino)butanoic acid | 5-[3-(Methylamino)propyl]-10,11-dihydro-5H-dibenzo[b,f]azepin-2-yl beta-D-glucopyranosiduronic acid |

|  | 1. **Cervical vs BEO** | 1. **Ovarian vs BEC** |
| --- | --- | --- |
| 1 | Cholic acid glucuronide | isopentenyl diphosphate |
| 2 | 9-[5-O-(Hydroxy{[hydroxy(phosphonooxy)phosphoryl]oxy}phosphoryl)pentofuranosyl]-6-methylene-6,9-dihydro-1H-purin-2-amine | 1-stearyl estercitric acid |
| 3 | D-Arginyl-L-histidyl-D-prolyl-D-tyrosine | Stearoylcarnitine |
| 4 | D-Isoleucyl-L-prolyl-L-tyrosyl-D-isoleucine | N-hexacosanoylsphingosine 1-phosphate |
| 5 | LTF4 | gln-asn |
| 6 | (10E,15Z)-9,12,13-Trihydroxy-10,15-octadecadienoic acid | 3,3'-[7,12-Bis(1-hydroxyethyl)-3,8,13,17-tetramethyl-2,18-porphyrindiyl]dipropanoic acid |
| 7 | 2-Docosahexaenoyl glycerol | 1-(1Z-hexadecenyl)-2-(8Z,11Z,14Z-icosatrienoyl)-sn-glycero-3-phosphoethanolamine |
| 8 | 1-Myristoyl-2-hydroxy-sn-glycero-3-PE | Stearoylcarnitine |
| 9 | 2-Hydroxy-3-[(9Z,12Z)-9,12-nonadecadienoyloxy]propyl (5Z,8Z,11Z,14Z)-5,8,11,14-icosatetraenoate | 1,2-dioleoyl-sn-glycero-3-phospho-(1'-sn-glycerol) |
| 10 | (2-Hydroxy-2-oxido-1,3,2-dioxaphospholan-4-yl)methyl palmitate | N(1)-acetylspermidine |
| 11 | 2,4-Dimethyl-3-pentanamine | N-(3-Carboxy-3-hydroxypropyl)-4-[(carboxymethyl)amino]threonine |
| 12 | 7a-Hydroxy-cholestene-3-one | Androsterone glucuronide |
| 13 | (2E,2'E)-3,3'-[2,4-Bis(1,3-benzodioxol-5-yl)-1,3-cyclobutanediyl]bis[1-(1-piperidinyl)-2-propen-1-one] | 7,9,12-Trihydroxy-2,2-dimethyl-2H,6H-pyrano[3,2-b]xanthen-6-one |
| 14 | 1-Stearoyl-2-arachidonoyl-sn-glycerol | Dehydroepiandrosterone sulfate |
| 15 | Ascorbyl palmitate | SM(d18:0/14:0) |
| 16 | Dehydroepiandrosterone sulfate | 4-({4,6-Dihydroxy-3-[(2E)-4-hydroxy-3-methyl-2-buten-1-yl]-2-methoxyphenyl}acetyl)-3-hydroxyphenyl hexopyranosiduronic acid |
| 17 | Cortisol | 2-[(11Z,14Z)-icosadienoyl]-sn-glycero-3-phosphoethanolamine |
| 18 | 5-Hydroxy-N-[(6-oxo-2-piperidinyl)methyl]-2-(2,2,2-trifluoroethoxy)benzamide | 18-Hydroxycortisol |
| 19 | 3,3'-[7,12-Bis(1-hydroxyethyl)-3,8,13,17-tetramethyl-2,18-porphyrindiyl]dipropanoic acid | Prostaglandin D2 Ethanolamide |
| 20 | 1-(1Z-hexadecenyl)-2-(8Z,11Z,14Z-icosatrienoyl)-sn-glycero-3-phosphoethanolamine | C20 Sphingomyelin (d18:1/20:0) |
| 21 | (4Z,7Z,10Z,19Z)-14-[(4Z,7Z,10Z,13Z,16Z,19Z)-4,7,10,13,16,19-Docosahexaenoyloxy]-4,7,10,12,16,19-docosahexaenoic acid | LysoPC(22:2(13Z,16Z)) |
| 22 | Ethyl 1,2,5,6-tetrahydro-1-methyl-3-pyridinecarboxylate | 4-[5,7-Dihydroxy-8-(3-methyl-2-buten-1-yl)-4-oxo-3,4-dihydro-2H-chromen-3-yl]-2-hydroxy-6-(3-methyl-2-buten-1-yl)phenyl hydrogen sulfate |
| 23 | (2R)-2-[(5Z,8Z,11Z,14Z,17Z)-5,8,11,14,17-Icosapentaenoyloxy]-3-(tetradecanoyloxy)propyl 2-(trimethylammonio)ethyl phosphate | 3-(2-Acetamidoethyl)-1-hexopyranuronosyl-5-methoxy-1H-indole |
| 24 | gln-asn | N-Heptadecanoylglycine |
| 25 | 1-[(9Z)-tetradecenoyl]-2-[(5Z,8Z,11Z,14Z)-icosatetraenoyl]-sn-glycerol | (7E)-3,8-Dimethyl-7-decen-1-yl trihydrogen diphosphate |
| 26 | (2R)-3-{[(2-Aminoethoxy)(hydroxy)phosphoryl]oxy}-2-[(9Z)-9-hexadecenoyloxy]propyl (9Z)-9-hexadecenoate | 2,4-Bis(3-methyl-2-buten-1-yl)-1,3,5-benzenetriol |
| 27 | 1-(Octadecyloxy)-3-(tetradecanoyloxy)-2-propanyl (6Z,9Z,12Z)-6,9,12-octadecatrienoate | N,N'-diacetylchitobiosyldiphosphodolichol |
| 28 | O-propenoyl-D-carnitine | (2R)-3-{[(2-Aminoethoxy)(hydroxy)phosphoryl]oxy}-2-[(1Z,11Z)-1,11-octadecadien-1-yloxy]propyl (15Z)-15-tetracosenoate |
| 29 | 3-(13-Hydroxytriacontyl)-5-methyl-2(5H)-furanone | Androstenedione |
| 30 | Dioleoylphosphatidylserine | 7Î±-Hydroxy-3-oxo-4-cholestenoic acid |
| 31 | N-Nonanoylglycine | 3-(Hexacosanoyloxy)-4-(trimethylammonio)butanoate |
| 32 | (7E)-3,8-Dimethyl-7-decen-1-yl trihydrogen diphosphate | (-)-Prostaglandin E2 |
| 33 | (5R,6S,7E,9E,11Z,14Z)-6-{[(2S)-2-Acetamido-2-carboxyethyl]sulfanyl}-5-hydroxy-7,9,11,14-icosatetraenoic acid | (2R)-1-(Icosanoyloxy)-3-(phosphonooxy)-2-propanyl 22-methyltetracosanoate |
| 34 | 2-Hydroxyestrone | 2-Methyl-3-(furfurylthio)pyrazine |
| 35 | 5,7,18-Trihydroxy-1,14,21,25-tetramethyl-4,20,23-trioxaheptacyclo[20.3.1.1~2,5~.0~3,18~.0~3,21~.0~6,15~.0~9,14~]heptacosa-8,11-diene-13,19,24,27-tetrone | Phosphoenolpyruvic acid |
| 36 | 3-Hydroxy-5, 8-tetradecadiencarnitine | 1,2,3-Trimyristoleoyl Glycerol |
| 37 | 2-Hydroxy-13,20-dimethoxy-4,7,17,22,22-pentamethyl-5,10,21,23-tetraoxahexacyclo[18.2.1.0~1,17~.0~4,16~.0~6,14~.0~8,12~]tricosa-6(14),7,12-trien-11-one | PC(o-16:1(9Z)/22:0) |
| 38 | 2-Hydroxy-2-(9-hydroxy-7-oxo-2,3-dihydro-7H-furo[3,2-g]chromen-2-yl)propyl hydrogen sulfate | 4-Hydroxyprolyltryptophan |
| 39 | S(8)-(2-methylbutanoyl)dihydrolipoamide | PC(o-20:1(11Z)/20:4(8Z,11Z,14Z,17Z)) |
| 40 | (6E,8R,10Z)-8-Hydroxy-3-oxo-6,10-hexadecadienoic acid | 1-(1Z-octadecenyl)-2-(4Z,7Z,10Z,13Z,16Z-docosapentaenoyl)-sn-glycero-3-phosphoethanolamine |
| 41 | 10-[4-(2,4,4-Trimethyl-2-pentanyl)phenoxy]-1-decanol | 2-[(2E)-3,7-Dimethyl-2,6-octadien-1-yl]-5-hydroxy-6-methoxy-3-methyl-1,4-benzoquinone |
| 42 | 1-Eicosapentaenoyl-2-docosahexaenoyl-sn-glycero-3-phosphoethanolamine | 1-oleoyl-2-arachidonoyl-sn-glycerol-3-phosphoethanolamine |
| 43 | N(1)-acetylspermidine | UROBILIN, (-)- |
| 44 | 4-O-beta-D-xylo-Hexopyranosyl-3-ulose-D-glucopyranose | (14R)-20-Amino-1-(3,4-dimethyl-5-pentyl-2-furyl)-17-hydroxy-17-oxido-11-oxo-12,16,18-trioxa-17lambda~5~-phosphaicosan-14-yl 13-(3,4-dimethyl-5-pentyl-2-furyl)tridecanoate |
| 45 | N-tricosanoylsphing-4-enine-1-phosphocholine | 1-(3,4-Dimethoxyphenyl)-3,5-decanediyl diacetate |
| 46 | C20 Sphingomyelin (d18:1/20:0) | 9H-Purin-6-amine, 9-[5-O-[hydroxy[[hydroxy[3-hydroxy-2,2-dimethyl-4-oxo-4-[[3-oxo-3-[[2-[[(3E,5Z,8Z)-1-oxo-3,5,8-tetradecatrien-1-yl]thio]ethyl]amino]propyl]amino]butoxy]phosphinyl]oxy]phosphinyl]-3-O -phosphono-beta-D-lyxofuranosyl]-, ion(4-) |
| 47 | 18-Hydroxycortisol | Lysophosphatidylethanolamine (22:6(4Z,7Z,10Z,13Z,16Z,19Z)/0:0) |
| 48 | (9cis)-O~15~-[(2S,3R,4R,5S,6R)-6-Carboxy-3,4,5-trihydroxytetrahydro-2H-pyran-2-yl]-2-oxoretinoic acid | Testosterone glucuronide |
| 49 | 2-trans,4-trans-Octadienoyl-CoA | [8-Formyl-5-methoxy-2-methyl-2-(4-methyl-2-oxo-3-penten-1-yl)-3,4-dihydro-2H-chromen-7-yl]methyl (9E,12E)-9,12-octadecadienoate |
| 50 | (3beta,4alpha,8alpha)-4,15-Diacetoxy-3-hydroxy-12,13-epoxytrichothec-9-en-8-yl propionate | 1-oleoyl-2-arachidonoyl-sn-glycero-3-phospho-L-serine |
| 51 | O-{[(2R)-3-[(7Z,10Z,13Z,16Z)-7,10,13,16-Docosatetraenoyloxy]-2-(tetracosanoyloxy)propoxy](hydroxy)phosphoryl}-L-serine | 1-(1Z-octadecenyl)-2-(9Z-octadecenoyl)-sn-glycero-3-phosphoethanolamine |
| 52 | 3b-Hydroxy-5-cholenoic acid | O-(Hydroxy{2-hydroxy-3-[(9E)-9-octadecenoyloxy]propoxy}phosphoryl)serine |
| 53 | 1-(Octadecyloxy)-3-(pentadecanoyloxy)-2-propanyl tetracosanoate | 7-Hydroxy-9-methoxy-6-(1,3,4-trihydroxy-2-butanyl)-1,2-dihydrocyclopenta[c]chromene-3,4-dione |
| 54 | N-Acetylserotonin sulfate | 1-Stearoyl-2-arachidonoyl-sn-glycerol |
| 55 | O-{[(2R)-2-[(4Z,7Z,10Z,13Z,16Z,19Z)-4,7,10,13,16,19-Docosahexaenoyloxy]-3-(tetradecanoyloxy)propoxy](hydroxy)phosphoryl}-L-serine | L-Hexanoylcarnitine |
| 56 | N~6~-[5-(1,2-Dithiolan-3-yl)pentanoyl]-D-lysine | (2R)-2-[(8Z,11Z,14Z)-8,11,14-Icosatrienoyloxy]-3-(pentadecanoyloxy)propyl 2-(trimethylammonio)ethyl phosphate |
| 57 | N~2~-(Carboxymethyl)arginine | 5-O-methyl embelin |
| 58 | (2R)-1-{[(2-Aminoethoxy)(hydroxy)phosphoryl]oxy}-3-hydroxy-2-propanyl (13Z,16Z)-13,16-docosadienoate | N-Acetyl-DL-histidine |
| 59 | (14R)-20-Amino-1-(3,4-dimethyl-5-pentyl-2-furyl)-17-hydroxy-17-oxido-11-oxo-12,16,18-trioxa-17lambda~5~-phosphaicosan-14-yl 13-(3,4-dimethyl-5-pentyl-2-furyl)tridecanoate | 3-[(6-Oxodecanoyl)oxy]-4-(trimethylammonio)butanoate |
| 60 | b-Sulfinyl pyruvate | 1-myristoyl-2-palmitoleoyl-sn-glycero-3-phosphocholine |
| 61 | (2R)-1-[(6Z,9Z,12Z)-6,9,12-Octadecatrienoyloxy]-3-(phosphonooxy)-2-propanyl (15Z)-15-tetracosenoate | 8-[(3,3-Dimethyl-2-oxiranyl)methyl]-7-methoxy-2H-chromen-2-one |
| 62 | Phosphoenolpyruvic acid | (2R)-1-(Icosanoyloxy)-3-(phosphonooxy)-2-propanyl (15Z)-15-tetracosenoate |
| 63 | 3-{[(2R)-1-Methyl-1-oxido-2-pyrrolidinyl]methyl}-5-[2-(phenylsulfonyl)ethyl]-1H-indole | Ketamine |
| 64 | 2-Amino-2-deoxy-alpha-D-glucopyranosyl-(1->4)-2-O-sulfo-alpha-L-idopyranuronosyl-(1->4)-2-deoxy-6-O-sulfo-2-(sulfoamino)-alpha-D-glucopyranose | 3b-Hydroxy-5-cholenoic acid |
| 65 | (3R,3aR,5S,5aR,5bR,9S,11aR,13aR,13bS)-5-Hydroxy-3a,5a,5b,8,8,11a-hexamethyl-3-(3-methyl-2-buten-1-yl)-1-oxooctadecahydro-1H-furo[3,4-c]naphtho[1,2-h]isochromen-9-yl 6-O-(6-deoxy-alpha-L-mannopyranosyl )-beta-D-glucopyranoside | 5,6-Dihydroxy-7-methoxy-2,2-dimethyl-3,4-dihydro-2H-chromen-4-yl hydrogen sulfate |
| 66 | 1-C-[(8E,10E)-35-Carboxy-28-hydroxy-5,7,9,19,29-pentamethyl-18,31-dioxo-13,17,38,39,40,41,42,43-octaoxaoctacyclo[31.4.1.1~1,35~.1~2,5~.1~20,24~.1~24,27~.1~29,32~.0~12,16~]tritetraconta-8,10-dien-14-yl ]-3,4-dideoxy-3-methylpentopyranose | Formiminoglutamic Acid |
| 67 | (2E)-[4-(3,4-Dihydroxyphenyl)-3-hydroxy-5-oxo-2(5H)-furanylidene](4-hydroxyphenyl)acetic acid | 2,4-dihydroxyheptadec-16-enyl acetate |
| 68 | (8E,15Z)-1,8,15-Heptadecatriene-11,13-diyne | Pentosidine |
| 69 | LysoPC(20:5(5Z,8Z,11Z,14Z,17Z)) | 2-Hydroxy-3-[(9Z,12Z)-9,12-nonadecadienoyloxy]propyl (15Z)-15-tetracosenoate |
| 70 | (5Z,17R)-23-Amino-20-hydroxy-20-oxido-14-oxo-15,19,21-trioxa-20lambda~5~-phosphatricos-5-en-17-yl (6Z,9Z,12Z,15Z)-6,9,12,15-octadecatetraenoate | N-[(15Z)-3-hydroxytetracos-15-enoyl]sphingosine-1-phosphocholine |
| 71 | 4-[(3E,5E,7E,9E,11E)-15-Hydroxy-3,7,12-trimethyl-3,5,7,9,11-pentadecapentaen-1-yn-1-yl]-3,5,5-trimethyl-3-cyclohexen-1-ol | 4-METHYL MEIQX |
| 72 | 6-{2-[(1E)-6-Amino-3-(3-amino-3-carboxypropyl)-6-carboxy-1-hexen-1-yl]-3,5-bis(3-amino-3-carboxypropyl)-1-pyridiniumyl}norleucine | 17-(Cyclopropylmethyl)-18-(2-hydroxy-3,3-dimethyl-2-butanyl)-6-methoxy-18,19-dihydro-4,5-epoxy-6,14-ethenomorphinan-3,10-diol |
| 73 | 1-hexadecanoyl-2-(5Z,8Z,11Z,14Z-icosatetraenoyl)-sn-glycero-3-phosphoethanolamine | 6-Methyl-7,8-dihydroimidazo[1,5-c]pyrimidine-5(6H)-thione |
| 74 | (DL)-3-O-Methyldopa | L-Cystine |
| 75 | 3-(2-Acetamidoethyl)-1-hexopyranuronosyl-5-methoxy-1H-indole | 4a-Hydroxy-12-methoxy-4,4,6a,8,13b-pentamethyl-1,4,4a,5,6,6a,9,13,13a,13b-decahydro-2H-benzo[a]furo[3,4-i]xanthene-3,11-dione |
| 76 | 1-[(9Z)-octadecenyl]-2-hexadecanoyl-sn-glycero-3-phosphocholine | N-Phenylacetylglutamic acid |
| 77 | LYSINOALANINE, (S,R) | 27-Nor-5Î²-cholestane-3Î±,7Î±,12Î±,24,25-pentol |
| 78 | 5-[(9-Hydroxy-8,8-dimethyl-2-oxo-9,10-dihydro-2H,8H-pyrano[2,3-f]chromen-10-yl)oxy]-8,8-dimethyl-2H,8H-pyrano[2,3-f]chromen-2-one | (8E,15Z)-1,8,15-Heptadecatriene-11,13-diyne |
| 79 | trimethylsilyl icosanoate | (2R)-2-[(7Z,10Z,13Z,16Z,19Z)-7,10,13,16,19-Docosapentaenoyloxy]-3-[(5Z,8Z,11Z,14Z,17Z)-5,8,11,14,17-icosapentaenoyloxy]propyl 2-(trimethylammonio)ethyl phosphate |
| 80 | (2R)-1-{[(2-Aminoethoxy)(hydroxy)phosphoryl]oxy}-3-hydroxy-2-propanyl (9Z,12Z,15Z)-9,12,15-octadecatrienoate | (2R)-1-[(2S,4R,5Z)-4-Benzyl-2,5-dihydroxy-5-{[(1R,2S)-2-hydroxy-2,3-dihydro-1H-inden-1-yl]imino}pentyl]-N-(2-methyl-2-propanyl)-2-piperazinecarboximidic acid (non-preferred name) |
| 81 | 1-oleoyl-2-arachidonoyl-sn-glycero-3-phospho-L-serine | PG(16:0/22:6(4Z,7Z,10Z,13Z,16Z,19Z)) |
| 82 | L-Tyrosyl-L-prolyl-D-glutaminyl-L-prolyl-L-glutaminyl-D-prolyl-D-phenylalanine | Tetraacetylethylenediamine |
| 83 | 1-[(6R)-2-Hydroxy-2-oxidotetrahydro-4H-furo[3,2-d][1,3,2]dioxaphosphinin-6-yl]-5-methyl-2,4(1H,3H)-pyrimidinedione | D-1-Piperideine-2-carboxylic acid |
| 84 | Cholesterol sulfate | 9H-Purin-6-amine, 9-[5-O-[hydroxy[[hydroxy[3-hydroxy-4-[[3-[[2-[[(3S,8E)-3-hydroxy-1-oxo-8-tetradecen-1-yl]thio]ethyl]amino]-3-oxopropyl]amino]-2,2-dimethyl-4-oxobutoxy]phosphinyl]oxy]phosphinyl]-3-O- phosphono-beta-D-lyxofuranosyl]-, ion(4-) |
| 85 | (2R)-1-[(Hydroxy{[(1s,3R)-2,3,4,5,6-pentahydroxycyclohexyl]oxy}phosphoryl)oxy]-3-(palmitoyloxy)-2-propanyl (9Z,12Z)-9,12-hexadecadienoate | 1-[(9Z)-tetradecenoyl]-2-[(5Z,8Z,11Z,14Z)-icosatetraenoyl]-sn-glycerol |
| 86 | sulfoglycochenodeoxycholic acid | Cholic acid glucuronide |
| 87 | Hept-2-ulose | (DL)-3-O-Methyldopa |
| 88 | Testosterone glucuronide | Methyl 4,9-dihydro-Î²-hydroperoxy-Î±-[(methoxycarbonyl)amino]-4,6-dimethyl-9-oxo-3H-imidazo[1,2-a]purine-7-butanoate |
| 89 | beta-D-Glucopyranuronosyl-(1->3)-(3xi)-2-acetamido-2-deoxy-beta-D-ribo-hexopyranosyl-(1->4)-beta-D-glucopyranuronosyl-(1->3)-(3xi)-2-acetamido-2-deoxy-beta-D-ribo-hexopyranose | 2,3-Dihydroxy-9-phenyl-2,3-dihydro-1H-phenalen-1-one |
| 90 | 2-Methyl-4-oxo-3-[(2E)-2-penten-1-yl]-2-cyclopenten-1-yl hexopyranoside | (-)-Prostaglandin E2 |
| 91 | 1-oleoyl-2-arachidonoyl-sn-glycerol-3-phosphoethanolamine | 3-[[O-D-Apio-Î²-D-furanosyl-(1â†’2)-O-[2-O-[(2E)-3-(4-hydroxy-3-methoxyphenyl)-1-oxo-2-propen-1-yl]-Î²-D-glucopyranosyl-(1â†’6)]-Î²-D-glucopyranosyl]oxy]-2-(3,4-dihydroxyphenyl)-5,7-dihydroxy-6-methoxy-4H-1-benzopyran-4-one |
| 92 | LysoPC(22:2(13Z,16Z)) | (2R)-1-[(Hydroxy{[(1S,5R)-2,3,4,6-tetrahydroxy-5-(phosphonooxy)cyclohexyl]oxy}phosphoryl)oxy]-3-(palmitoyloxy)-2-propanyl (13Z,16Z)-13,16-docosadienoate |
| 93 | 2,3,23-Trihydroxyurs-12-en-28-oic acid | (4S)-4-cyclohexyl-1-({[2-methyl-1-(propanoyloxy)propoxy](4-phenylbutyl)phosphoryl}acetyl)-L-proline |
| 94 | Serotonin | 1-(1Z-octadecenyl)-2-(4Z,7Z,10Z,13Z,16Z,19Z-docosahexaenoyl)-sn-glycero-3-phosphocholine |
| 95 | 2-[(1Z,11Z)-1,11-Octadecadien-1-yloxy]-3-[(9Z)-9-tetradecenoyloxy]propyl 2-(trimethylammonio)ethyl phosphate | 12-Methoxy-4,4,6a,8,13b-pentamethyl-1,4,4a,5,6,6a,9,13,13a,13b-decahydro-2H-benzo[a]furo[3,4-i]xanthene-3,11-dione |
| 96 | 8-Hydroxyhexadecanedioic acid | 1-alpha,24R,25-Trihydroxyvitamin D2 |
| 97 | O-{[(2R)-2-[(13Z)-13-Docosenoyloxy]-3-(pentadecanoyloxy)propoxy](hydroxy)phosphoryl}-L-serine | PC(o-18:0/18:2(9Z,12Z)) |
| 98 | N-(4-Carbamimidamidobutyl)-5-{(1Z)-3-[(4-carbamimidamidobutyl)amino]-3-oxo-1-propen-1-yl}-7-hydroxy-2-(4-hydroxyphenyl)-2,3-dihydro-1-benzofuran-3-carboxamide | Dioleoylphosphatidylserine |
| 99 | 1,3-Bis(tetradecanoyloxy)-2-propanyl (5Z,8Z,11Z,14Z,17Z)-5,8,11,14,17-icosapentaenoate | (2R)-3-(Phosphonooxy)-2-(tetradecanoyloxy)propyl (9Z)-9-tetradecenoate |
| 100 | N-Acetyl-5-oxo-L-norvaline | O-pentadecanoylcarnitine |

**Table S3:** Top 100 metabolites were identified and ranked according to the weightage for **A) BECO vs Normal B) Breast vs ECO C) Endometrial vs BCO D) Cervical vs BEO E) Ovarian vs BEC.**
